# SanA Plays a Role in Peptidoglycan Integrity in *Escherichia coli*

**DOI:** 10.1111/mmi.70058

**Published:** 2026-02-20

**Authors:** Honoka Yamaguchi, Risa Ago, Yuhei O. Tahara, Mari Inoue, Hironori Niki, Makoto Miyata, Daisuke Shiomi

**Affiliations:** ^1^ Department of Life Science, College of Science Rikkyo University Toshima Tokyo Japan; ^2^ Graduate School of Science Osaka Metropolitan University Osaka Japan; ^3^ The OMU Advanced Research Center for Natural Science and Technology Osaka Metropolitan University Osaka Japan; ^4^ Microbial Physiology Laboratory, Department of Gene Function and Phenomics National Institute of Genetics Mishima Shizuoka Japan; ^5^ Department of Genetics The Graduate University for Advanced Studies SOKENDAI Mishima Shizuoka Japan

**Keywords:** *E. coli*, peptidoglycan, Rod complex

## Abstract

Peptidoglycan synthesis and degradation are both essential for bacterial growth, and damaged peptidoglycan must be continuously repaired. In *Escherichia coli* , peptidoglycan required for cell elongation is synthesized by the Rod complex. Although RodZ is a non‐essential component of this complex, its dysfunction leads to aberrant peptidoglycan synthesis, resulting in defects in cell shape and impaired growth. We previously isolated several suppressor mutants that restore growth in cells with impaired RodZ function (RMR cells). Most suppressor mutations mapped to components in the Rod complex other than RodZ. However, one suppressor mutation was identified in *sanA*, a gene not previously associated with the Rod complex. This mutation, *sanA* ^*M27R*^, represents a loss‐of‐function allele. Here, we show that SanA is associated with PBP1B, a non‐essential yet physiologically important peptidoglycan synthase. Loss of SanA function partially restored the growth of RMR cells, accompanied by enhanced peptidoglycan synthesis and alleviation of structural defects in the cell wall. These findings indicate that SanA contributes to the regulation of peptidoglycan synthesis and cell wall integrity, potentially through functional interplay with PBP1B‐dependent pathways.

## Graphical abstract

SanA is a novel regulator of peptidoglycan synthesis, potentially through functional interplay with PBP1B‐dependent pathways.

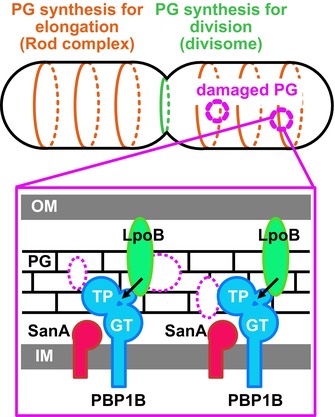

## 1 Introduction

Most bacteria are covered with peptidoglycan, a large molecule composed of alternating sugar chains of *N*‐acetylglucosamine and *N*‐acetylmuramic acid, cross‐linked by short peptides, typically 4–5 amino acids long (Egan et al. 2020; Rohs and Bernhardt 2021; Galinier et al. 2023). Proper synthesis and maintenance of peptidoglycan are important for bacterial morphogenesis and viability. Thus, peptidoglycan and its synthetic pathway are targets of several antibiotics such as penicillin. The key proteins to synthesize peptidoglycan are penicillin‐binding proteins (PBPs) (Sauvage et al. 2008). PBPs are divided into two classes: high molecular weight (HMW) PBPs and low molecular weight (LMW) PBPs. HMW PBPs are further divided into two classes (class A and class B). In *Escherichia coli* , class A PBPs (PBP1A encoded by *mrcA* gene and PBP1B encoded by *mrcB*) have both glycosyl‐transferase (GTase) and transpeptidase (TPase) activities while class B PBPs (PBP2 encoded by *pbpA* and PBP3 encoded by *ftsI*) have only a TPase activity. Therefore, PBP2 and PBP3 function with RodA and FtsW which have GTase activity, respectively (Rohs and Bernhardt 2021). Peptidoglycan synthesis during the elongation and division of cells is regulated by the Rod and divisome complexes, respectively (Egan et al. 2020; Rohs and Bernhardt 2021; Galinier et al. 2023). The Rod complex consists of various proteins, such as bacterial actin MreB, a transpeptidase PBP2, a transglycosylase RodA, inner membrane proteins MreC, MreD, and RodZ, whereas the divisome complex consists of various proteins, such as bacterial tubulin FtsZ, bacterial actin FtsA, a transpeptidase PBP3, and a transglycosylase FtsW. Class A PBPs which have both GTase and TPase activities are included in the complexes. It has been shown that PBP1A can be associated with the Rod complex, whereas PBP1B can be associated with the divisome (Bertsche et al. 2006; Müller et al. 2007; Banzhaf et al. 2012; Boes et al. 2021). While single‐gene deletion mutants of either *mrcA* (encoding PBP1A) or *mrcB* (encoding PBP1B) are viable, simultaneous deletion of both genes is synthetically lethal (Yousif et al. 1985). These findings suggest that the two proteins are functionally compatible and that their roles are at least partially redundant. To maintain peptidoglycan integrity, cells must repair it upon damage when exposed to various stresses such as extreme pH. Under stress, peptidoglycan is repaired by PBP1A and PBP1B (Vigouroux et al. 2020) which are activated by the outer membrane lipoproteins LpoA and LpoB, respectively (Paradis‐Bleau et al. 2010; Typas et al. 2010), and _LD_‐transpeptidase (LDTs) (Morè et al. 2019; Aliashkevich and Cava 2022). Compared to PBP1A, PBP1B seems to play a more important role. For example, the loss of PBP1B has a greater impact on cell stiffness (Auer et al. 2016) and results in a slower rate of peptidoglycan insertion (Caparrós et al. 1994). Therefore, even without evident stress, peptidoglycan must be repaired mainly by the PBP1B‐containing complex.

In *E. coli* , 90%–98% of the crosslinks in peptidoglycan are 4–3 cross‐links (between _D_‐Ala and *meso*‐diaminopimelic acid in adjusting peptides) and 2%–10% are 3–3 cross‐links (between two *meso*‐diaminopimelic acids in adjusting peptides) (Höltje and Schwarz 1988; Höltje 1998; Morè et al. 2019). The 4–3 cross‐links are usually formed by PBPs in the Rod complex, divisome, and aPBPs, whereas the 3–3 crosslinks are formed by LDTs, which have a structure different from that of PBPs. PBP1B (GTase), LpoB, PBP6a, and LdtD are required for the repair of peptidoglycan defects caused by abnormal LPS transport in LptC‐depleted cells (Morè et al. 2019). Thus, peptidoglycan crosslinking and the proteins involved differ during peptidoglycan synthesis and repair.

In nutrient‐rich media, several components of the Rod complex such as MreB are essential for growth (Bendezú and de Boer 2008); however, RodZ is a nonessential protein (Shiomi et al. 2008; Bendezú et al. 2009). Cells producing the Rod complex lacking *rodZ* or producing a chimeric protein that replaces the transmembrane region of RodZ with that of MalF (hereafter, referred to as RMR) lose their rod shape and show a slower growth rate (Shiomi et al. 2008; Bendezú et al. 2009; Ago et al. 2023). We purified peptidoglycan from wild‐type (WT), ∆*rodZ*, and RMR cells and found that ∆*rodZ* and RMR cells have several large holes in the peptidoglycan compared with that in WT cells (Ago et al. 2023). The results indicate that the Rod complex activity is reduced in these mutant cells, resulting in incorrect peptidoglycan synthesis. Furthermore, we isolated several suppressor strains with restored growth rates from ∆*rodZ* and RMR cells (Shiomi et al. 2013; Ago et al. 2023). Peptidoglycan purified from these suppressor mutants exhibited decreased hole size and number. Mutations in the components of the Rod complex such as MreB, PBP2 encoded by *mrdA*, and RodA encoded by *mrdB* were found to be the suppressor mutations. Most of these mutations occur at the protein–protein interaction interfaces. Therefore, RodZ may regulate the interactions between proteins in the Rod complex. Apparently, these suppressor mutations restored protein–protein interactions and, thereby, restored the activity of the Rod complex. Interestingly, one of the suppressor mutants isolated from the cells producing RMR was found to be mutated in a factor not previously known to be present in the Rod complex. The gene was *sanA*.

SanA suppressed vancomycin‐sensitive strains when overexpressed and ∆*sanA* cells became vancomycin‐sensitive at 43°C (Rida et al. 1996). Vancomycin is generally less effective against Gram‐negative bacteria, including *E. coli* , because of its low outer membrane permeability. Although the molecular mechanism behind the vancomycin‐sensitivity of ∆*sanA* cells is unknown, vancomycin susceptibility of ∆*sanA* cells might be due to the brittleness of the outer membrane, which increases vancomycin permeability. However, two different results have been reported regarding this possibility (Paradis‐Bleau et al. 2014; Aleksandrowicz et al. 2024). Paradis‐Bleau et al. reported that the surface structure of ∆*sanA* cells was not significantly altered because their membrane permeability was similar to that of WT cells at 30°C (Paradis‐Bleau et al. 2014), whereas Aleksandrowicz et al. reported that SanA is responsible for membrane integrity (Aleksandrowicz et al. 2024). Aleksandrowicz et al. determined the membrane integrity at 37°C by measuring the influx of a cationic dye in the presence of CCCP, which is a protonophore that prevents the efflux of the dye by active pumps. Therefore, treatment with CCCP may affect the activity of many membrane proteins. For example, CCCP inhibits the MurJ activity, which is required to flip Lipid II (Rubino et al. 2018). Thus, the peptidoglycan that supports the outer membrane from the periplasmic side in the cells may be weakened in the presence of CCCP. The peptidoglycan layer and the outer membrane are physically linked by Lpp. Thus, alterations in peptidoglycan integrity can disrupt this linkage. Consistent with this, it has been reported that an Lpp variant (Lpp^+21^) exhibits increased susceptibility to vancomycin (Mathelié‐Guinlet et al. 2020). Therefore, it is thought that the permeability of vancomycin may be increased by alternation of peptidoglycan. Thus, the effect of ∆*sanA* cells on the vancomycin resistance and the membrane permeability remains controversial. While we were preparing this manuscript, Gundavarapu et al., reported that SanA is a novel regulatory protein of peptidoglycan synthesis (Gundavarapu et al. 2025). In addition, the authors found that peptidoglycan synthesis was increased in cells lacking *sanA* while the chemical structure of peptidoglycan (i.e., the contents of 4–3 and 3–3 cross‐linked peptides) was comparable in WT and ∆*sanA*, implying that SanA negatively regulates peptidoglycan synthesis in WT cells. A preprint of another paper on SanA has been published on bioRxiv (Carr et al. 2025). In the preprint, it is suggested that the deletion of *sanA* (and *wecA*) may result in an excess supply of Lipid II. The authors speculate that SanA might regulate the availability of Lipid II for septal peptidoglycan synthesis. However, since interacting partners of SanA were not identified in those papers, the molecular mechanism behind the regulation of peptidoglycan synthesis by SanA is still unclear.

In this study, we investigated the relationship between SanA and PBP1B, a class A PBP involved in peptidoglycan synthesis and repair, and suggest that SanA plays a role in peptidoglycan integrity.

## 2 Results and Discussion

### 2.1 Isolation of *sanA* Mutant

**FIGURE 1:**
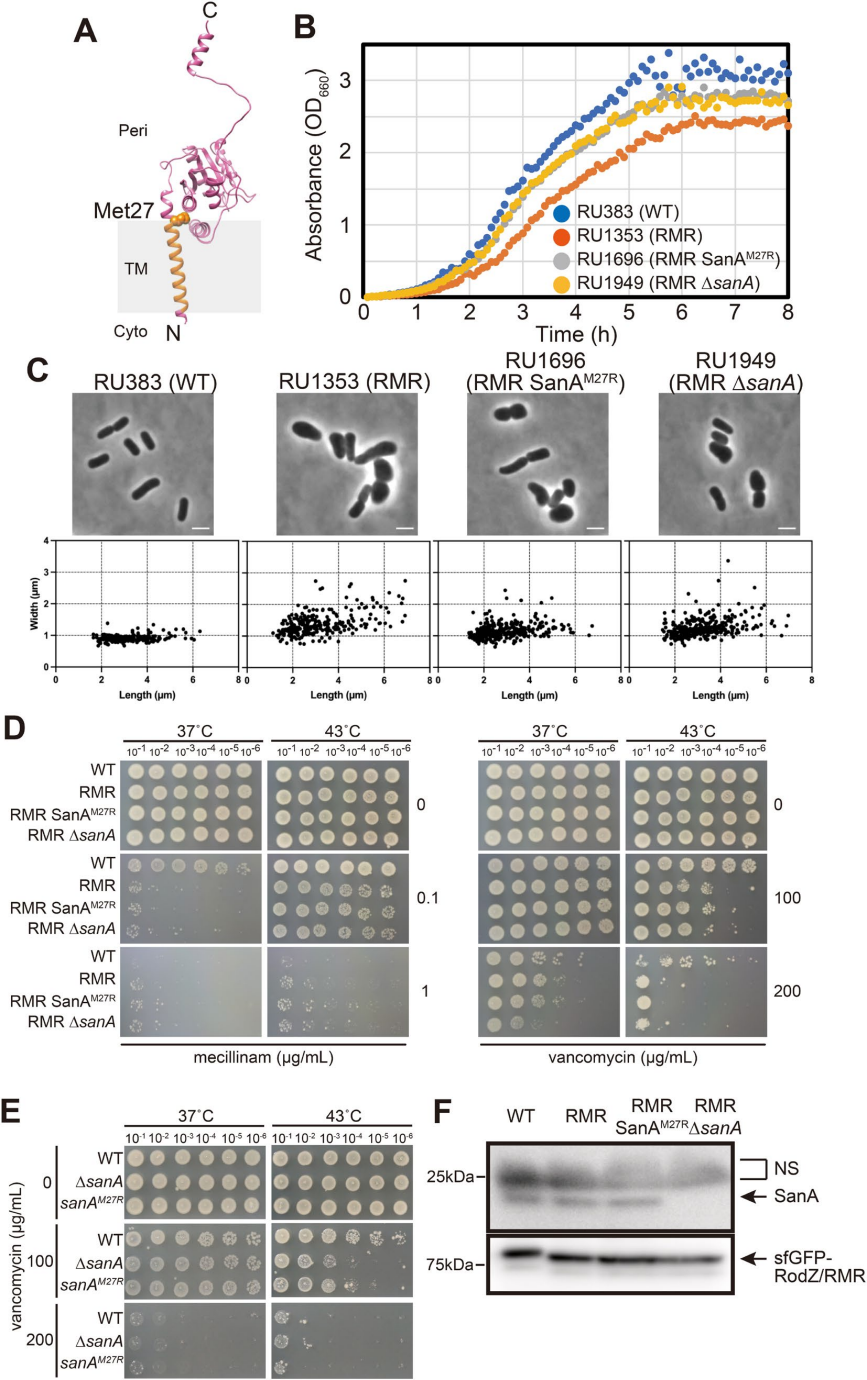
Characterization of SanA^M27R^ mutant. (A) Predicted structure of SanA. The three‐dimensional structure predicted using Alphafold2 was retrieved from a database (https://alphafold.ebi.ac.uk). Met27 is shown. Cyto; cytoplasmic domain, TM; transmembrane domain, Peri; periplasmic domain. (B) Growth curve of RU383 (WT; sfGFP‐RodZ), RU1353 (RMR; sfGFP‐RMR), RU1696 (RMR SanA^M27R^), and RU1949 (sfGFP‐RMR ∆*sanA*) cells. The cells were cultured in L medium at 37°C and their absorbance (OD_660_) was recorded every 5 min. (C) Morphology of RU383 (WT; sfGFP‐RodZ), RU1353 (RMR; sfGFP‐RMR), RU1696 (sfGFP‐RMR SanA^M27R^), and RU1949 (sfGFP‐RMR ∆*sanA*) cells cultured in L medium to log phase at 37°C. The graph plots the length and width of individual cells. Scale bars: 2 μm. (D) Mecillinam or vancomycin sensitivity of RU383 (WT; sfGFP‐RodZ), RU1353 (RMR; sfGFP‐RMR), RU1696 (sfGFP‐RMR SanA^M27R^), and RU1949 (sfGFP‐RMR ∆*sanA*) cells. The cells were cultured overnight in L medium at 37°C and serially diluted in fresh medium. Diluted cells were spotted onto L plates containing mecillinam or vancomycin and incubated at 37°C or 43°C for 24 h. (E) Vancomycin‐sensitivity of RU1651 (WT), RU1865 (∆*sanA*), and RU1724 (SanA^M27R^) cells. The cells were cultured overnight in L medium at 37°C and serially diluted in fresh medium. Diluted cells were spotted onto L plate containing vancomycin and incubated at the indicated temperature for 24 h. (F) Immunoblot of RU383 (WT; sfGFP‐RodZ), RU1353 (RMR; sfGFP‐ RMR), RU1696 (sfGFP‐RMR SanA^M27R^), and RU1949 (sfGFP‐RMR ∆*sanA*) cells with the anti‐SanA (top) or anti‐RodZ antibodies (bottom). NS, non‐specific band reacting with anti‐SanA antibody.

We previously isolated suppressor mutants of the slow‐growth phenotype of RU1353 cells producing the sfGFP‐RMR protein, a super‐folder GFP (sfGFP) fused with a mutant RodZ in which the transmembrane (TM) domain of RodZ was replaced with the first transmembrane (TM1) domain of MalF (Ago et al. 2023). All the suppressor strains, except for one, carried a mutation in a component of the Rod complex, such as MreB or PBP2. These mutations suppressed the reduced peptidoglycan synthesis activity of the Rod complex containing RMR. Mutations other than those in the Rod complex occurred in *sanA*, yielding a variant that produced the SanA^M27R^ protein. SanA was predicted to be a transmembrane protein composed of 239 amino acids, with a transmembrane domain from Val5 to Met27 (Figure 1A). Therefore, Met27 lies at the boundary between the TM and periplasmic domains. The facts that overproduction of SanA complements a vancomycin‐sensitive mutant and ∆*sanA* cells are sensitive to vancomycin at 43°C (Rida et al. 1996) suggest that SanA plays a role in the biogenesis of the cell envelope. Possibly, the permeability of the outer membrane is altered in ∆*sanA* cells, which enables the penetration of vancomycin into the periplasmic domain although the two contradict results have been shown regarding this possibility (Paradis‐Bleau et al. 2014; Aleksandrowicz et al. 2024). We examined membrane permeability of WT and ∆*sanA* cells using a CPRG (Chlorophenol Red‐β‐D‐galactopyranoside) assay (Paradis‐Bleau et al. 2014). We included ∆*elyC* cells as a control in this assay. The assay was performed at three different temperatures (30°C, 37°C, and 43°C), since previous studies had conducted it at 30°C or room temperature, whereas the phenotype associated with ∆*sanA* was observed specifically at 43°C. As reported previously, ∆*elyC* cells formed red to purple colonies at 30°C (the CPRG^+^ phenotype), while wild‐type and ∆*sanA* cells remained white (Figure S1), indicating that only the surface layer of the ∆*elyC* mutant is defective. Surprisingly, at 37°C, all three strains exhibited a faint red coloration. At 43°C, wild‐type and ∆*sanA* cells turned red to purple, whereas ∆*elyC* cells showed the same faint red phenotype observed at 37°C. Although these results are difficult to interpret, one point is clear: wild‐type and ∆*sanA* cells displayed the same phenotype under these conditions, while ∆*elyC* cells exhibited a distinct phenotype. This suggests that the membrane permeability of wild‐type and ∆*sanA* cells is comparable, consistent with the conclusions of Paradis‐Bleau et al. (Paradis‐Bleau et al. 2014). We next examined the susceptibility of WT and ∆*sanA* strains to several antibiotics (Figure S2). We found that only vancomycin susceptibility was altered by SanA deficiency while susceptibility to other antibiotics was unaffected. If SanA levels indeed modulate the membrane integrity, changes in susceptibility to antibiotics other than vancomycin would also be expected. In Gram‐negative bacteria, increased vancomycin susceptibility is generally attributed to defects in outer membrane permeability. However, our permeability assays indicate that deletion of *sanA* does not measurably alter outer membrane permeability (Figure S1). Thus, the vancomycin phenotype observed in ∆*sanA* cells cannot be readily explained by changes in outer membrane permeability alone. We also examined the susceptibility of WT cells overproducing SanA to mecillinam, a specific inhibitor of PBP2, and aztreonam, a specific inhibitor of PBP3, as well as vancomycin. It should be emphasized that this level of SanA protein (induced with 0.1 mM IPTG) does not inhibit the proliferation of WT cells. Overproduction of SanA conferred resistance to vancomycin as previously shown (Rida et al. 1996). In contrast, SanA overproduction did not affect susceptibility to mecillinam, and the cells appeared only slightly more sensitive to aztreonam, although the difference, if any, was marginal (Figure S3). Given that Gundavarapu et al. reported that SanA inhibits peptidoglycan synthesis during cell division (Gundavarapu et al. 2025), increased susceptibility to aztreonam due to SanA overproduction would not be contradictory to their findings. Considering that *sanA* was found to be a suppressor mutation in the peptidoglycan synthesis complex (the Rod complex), which has a reduced peptidoglycan synthesis activity (Ago et al. 2023), and that SanA is involved in vancomycin resistance (Rida et al. 1996), SanA is likely to be a novel factor involved in the synthesis and maintenance of peptidoglycan. The growth rate and shape of ∆*sanA* cells were comparable with those of WT cells (Figure S4), which indicates that SanA is not directly involved in the regulation of growth rate and cell shape. These results are consistent with the results shown by Gundavarapu et al. (2025).

### 2.2 Characterization of *sanA* Mutant

To characterize the *sanA* ^*M27R*^ mutation, we reintroduced it in RU1353 (sfGFP‐RMR) to obtain RU1696 (sfGFP‐RMR *sanA* ^*M27R*^). We also constructed the sfGFP‐RMR ∆*sanA* strain (RU1949) for comparison. We characterized the growth of WT (RU383: sfGFP‐RodZ), RMR (RU1353: sfGFP‐RMR), the suppressor strain (RU1696: sfGFP‐RMR *sanA* ^*M27R*^), and sfGFP‐RMR ∆*sanA* strain as a control (RU1949: sfGFP‐RMR ∆*sanA*). The growth of RU1353 (RMR) (doubling time: 36.0 min) was significantly slower than that of other strains. RU1696 (sfGFP‐RMR *sanA* ^*M27R*^) (doubling time: 33.0 min) cells grew faster than RU1353 cells, but slower than RU383 (WT) cells (doubling time: 30.1 min) (Figure 1B), indicating that SanA^M27R^ functions as a suppressor of RMR cells, but only partially suppresses the growth defect. The doubling time of RU1949 (RMR ∆*sanA*) (doubling time: 32.4 min) was comparable to that of RU1696 (sfGFP‐RMR *sanA* ^*M27R*^) (doubling time: 33.0 min). We also examined the cell shape of these strains (Figure 1C). As shown previously (Ago et al. 2023), RMR cells exhibited abnormal shapes. Suppressor mutants carrying mutations in the Rod complex components, such as MreB^A125V^, recovered the slow growth rate of RMR cells and their morphological abnormalities (Ago et al. 2023). RMR cells producing SanA^M27R^ were not rod‐shaped. The length of RMR cells producing SanA^M27R^ was not significantly different from that of RMR cells, whereas the width of RMR cells producing SanA^M27R^ was significantly thinner and thicker than that of RMR and WT cells, respectively (Figure 1C, Table 1). These results indicate that SanA^M27R^ can suppress the slow growth of RMR, at least partially, and reduce the cell width but not the length. Furthermore, although RMR cells producing MreB^A125V^ were resistant to mecillinam (Ago et al. 2023), neither the SanA^M27R^ mutation nor deletion of *sanA* conferred mecillinam resistance to RMR cells (Figure 1D). These phenotypes are clearly different from that of other RMR suppressor mutations such as MreB^A125V^. The RMR suppressors found thus far in the Rod complex components suppress both the growth rate and cell morphology (both the length and width) (Ago et al. 2023), whereas the SanA^M27R^ mutation restores only the growth rate, and the effect is partial. We also examined whether the SanA^M27R^ mutant could restore the Rod complex formation. The SanA^M27R^ mutant failed to restore the formation of the complex (Figure S5). These results indicate that the suppression mechanism of SanA^M27R^ is different from that of MreB^A125V^. The suppressor mutations identified thus far compensate for the reduced elongation activity caused by the Rod complex containing RMR (Ago et al. 2023), whereas the SanA^M27R^ mutation compensates for an activity or phenotype other than the elongation activity of the Rod complex containing RMR. RMR exhibited higher sensitivity to vancomycin than the wild type; however, no difference in sensitivity was observed depending on the presence or absence of the *sanA* ^*M27R*^ mutation (Figure 1D). Interestingly, the growth and cell shape of RU1696 (RMR *sanA* ^*M27R*^) were comparable to those of RU1949 (RMR ∆*sanA*) (Figure 1B,C, Table 1), suggesting that the M27R mutation is a loss‐of‐function mutation in SanA. If this is true, cells producing SanA^M27R^ should be sensitive to vancomycin similarly to ∆*sanA* cells. Cells producing SanA^M27R^ and cells lacking *sanA* were indeed more sensitive to vancomycin than the WT cells (Figure 1E). The M27R mutation possibly decreases the stability of SanA so that the mutant behaves like ∆*sanA* cells. Immunoblotting using an anti‐SanA antibody revealed that SanA^M27R^ was as stable as WT SanA (Figure 1F) and the result suggests that SanA^M27R^ is inserted in the inner membrane in the same manner as WT. These results suggest that SanA^M27R^ is a loss‐of‐function protein.

**TABLE 1:** Length and width of each strain.

Strain	Length^a^ (μm)	*N* ^b^	*p* value^c^
RU383 (WT)	3.07 ± 0.90	297	NA
RU1353 (RMR)	3.11 ± 1.27	273	0.6205 (vs. WT)
RU1696 (RMR *sanA* ^*M27R*^)	2.90 ± 1.02	341	0.0316 (vs. WT), 0.0231 (vs. RMR)
RU1949 (RMR ∆*sanA*)	3.25 ± 1.09	320	0.0251 (vs. WT), 0.1619 (vs. RMR)

Strain	Width^a^ (μm)	*N* ^b^	*p* value^c^
RU383 (WT)	0.95 ± 0.21	297	NA
RU1353 (RMR)	1.39 ± 0.35	273	< 0.0001 (vs. WT)
RU1696 (RMR *sanA* ^*M27R*^)	1.17 ± 0.27	341	< 0.0001 (vs. WT), < 0.0001 (vs. RMR)
RU1949 (RMR ∆*sanA*)	1.25 ± 0.30	320	< 0.0001 (vs. WT), < 0.0001 (vs. RMR)

^a^ Mean length and width ± standard deviation (SD) are shown.

^b^ Nubmers of cells counted are shown.

^c^ *p* values were determined by unpaired *t*‐test. Statistical significance was defined as *p* < 0.05.

We also examined whether the SanA^M27R^ mutation or deletion of the *sanA* gene could rescue the slow‐growth phenotype of ∆*rodZ* cells. Both the SanA^M27R^ mutation and *sanA* deletion partially alleviated the slow‐growth phenotype of ∆*rodZ* cells, although growth did not recover to the level of the wild‐type strain (Figure S6A). We also observed cell morphology of those cells. The length and width of ∆*rodZ sanA* ^*M27R*^ and ∆*rodZ* ∆*sanA* cells were significantly shorter and thinner than those of ∆*rodZ* cells but longer and thicker than those of WT cells (Figure S6B, Table S1). Taken together, these results indicate that the SanA^M27R^ mutation and *sanA* deletion partially suppress the growth defect caused by *rodZ* deficiency but are insufficient to correct the morphological abnormality. The cell length of the *sanA* ^*M27R*^ and ∆sanA cells was significantly longer than that of WT cells, although the difference was rather subtle.

Thus, the *sanA* ^*M27R*^ mutation and deletion of *sanA* may cause a slight increase in cell length, but the effect appears to be minimal. In fact, Gundavarapu et al. reported that there was no appreciable difference in cell length between the wild type and the *sanA* mutant when grown on LB medium (Gundavarapu et al. 2025). It should be noted, however, that our LB medium contained 0.5% NaCl, whereas theirs contained 1% NaCl. However, since both the *sanA* ^*M27R*^ mutation and *sanA* deletion function, at least partially, as suppressors of ∆*rodZ* or RMR cells, whose cell‐elongation activity is impaired, it is conceivable that *sanA* loss may slightly enhance cell‐elongation ability. We then examined the sensitivities to mecillinam and aztreonam, a specific inhibitor of PBP3 (FtsI) (Figure S7). The *sanA*‐deficient cells (*sanA* ^*M27R*^ and ∆*sanA*) exhibited mecillinam susceptibility equivalent to that of WT cells but displayed a slight increase in resistance to aztreonam compared with WT cells. This observation is consistent with the report by Gundavarapu et al., which showed an improvement in colony‐forming ability in the *sanA*‐deleted *ftsI23* mutant strain (Gundavarapu et al. 2025). This is also consistent with our observation that overexpression of SanA resulted in a slight increase in susceptibility to aztreonam (Figure S2).

### 2.3 SanA Function Is Associated with PBP1B/LpoB

**FIGURE 2:**
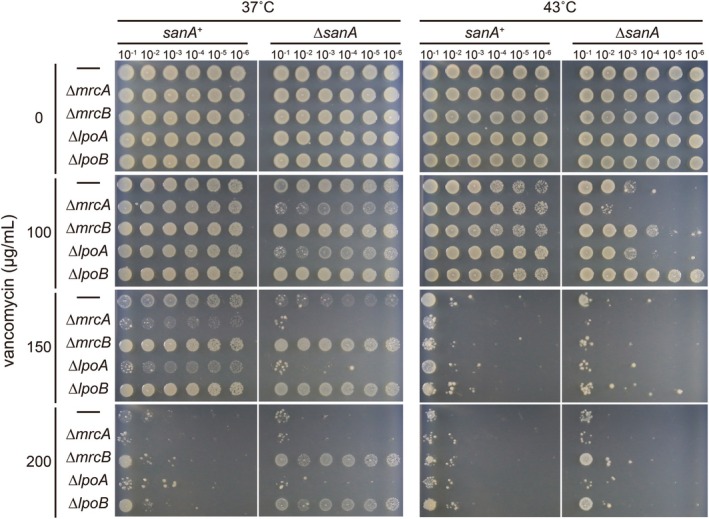
Vancomycin‐sensitivity of cells lacking PBPs and SanA. BW25113 (WT), RU1934 (∆*sanA*), RU1998 (∆*mrcA*), RU1999 (∆*mrcB*), RU2110 (∆*mrcA* ∆*sanA*), RU2112 (∆*mrcB* ∆*sanA*), and RU2208 (∆*lpoA*), RU2209 (∆*lpoB*), RU2210 (∆lpoA ∆*sanA*), and RU2211 (∆*lpoB* ∆*sanA*) cells were cultured overnight in L medium at 37°C and serially diluted in fresh medium. The diluted cells were spotted onto L plate containing vancomycin and incubated at 37°C or 43°C for 24 h.

Because loss of SanA function partially restores growth in RodZ‐impaired cells and simultaneously increases susceptibility to vancomycin, we hypothesized that SanA may influence processes related to peptidoglycan synthesis. Vancomycin binds to the D‐Ala‐D‐Ala moiety of nascent peptidoglycan (Reynolds 1989), which is utilized as a substrate during polymerization and crosslinking reactions. These reactions are mediated by penicillin‐binding proteins (PBPs) that act directly on nascent peptidoglycan, including both class A and class B PBPs. Because the class B PBPs PBP2 and PBP3 are essential for cell elongation and division, respectively, and therefore cannot be genetically deleted, we focused our analysis on the major class A PBPs, PBP1A and PBP1B. PBP1A interacts with PBP2 (Banzhaf et al. 2012), which is a component of the Rod complex, and PBP1B is partially interchangeable with PBP1A. However, double mutant cells lacking both PBP1A and PBP1B are synthetically lethal (Kato et al. 1985). Therefore, we first investigated whether SanA is functionally associated with PBP1A and/or PBP1B. We constructed double mutants of *sanA* and *mrcA* (encoding PBP1A) and *sanA* and *mrcB* (encoding PBP1B) and examined their vancomycin sensitivity (Figure 2). The sensitivity of ∆*mrcA* cells to vancomycin was relatively similar to that of ∆*sanA* cells (37°C, vancomycin_150 μg/mL) and ∆*mrcA* and ∆*sanA* exhibited synergistic effects on viability (43°C, vancomycin_100 μg/mL). These results suggest that PBP1A, like SanA, is involved in vancomycin resistance, and that these proteins likely contribute independently. However, ∆*mrcB* cells were more resistant to vancomycin than WT cells (37°C, vancomycin_150, 200 μg/mL), indicating that PBP1B contributes to vancomycin resistance in a manner opposite to that of PBP1A. ∆*mrcB ∆sanA* cells showed greater resistance to vancomycin than ∆*mrcB* cells (37°C, vancomycin_150, 200 μg/mL). Although the interpretation of these results is challenging, they suggest that the function of SanA could be more closely associated with PBP1B than that of PBP1A. PBP1A and PBP1B activities are regulated by the lipoproteins LpoA and LpoB, respectively (Paradis‐Bleau et al. 2010; Typas et al. 2010). Next, we constructed double mutants of *sanA* and *lpoA*, and *sanA* and *lpoB*. The vancomycin sensitivities of ∆*lpoA* and ∆*sanA* ∆*lpoA* cells were the same as those of ∆*mrcA* and ∆*sanA* ∆*mrcA* cells whereas the sensitivities of ∆*lpoB* and ∆*sanA* ∆*lpoB* cells were the same as those of ∆*mrcB* and ∆*sanA* ∆*mrcB* cells (Figure 2). These results suggest that SanA is more closely linked to PBP1B/LpoB than to PBP1A/LpoA with respect to vancomycin‐related phenotypes.

### 2.4 Interaction Between SanA and PBP1B

**FIGURE 3:**
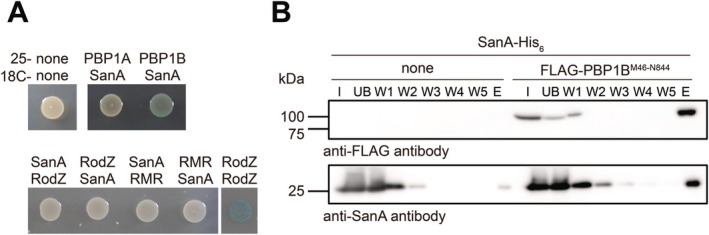
Interaction between SanA and PBP1B. (A) Interaction of SanA with PBP1A, PBP1B, RodZ, and RMR was examined using BACTH. DHM1 cells carrying the indicated plasmids were spotted on L plate containing X‐gal and the plate was incubated at 30°C for 24 h. Self‐interaction of RodZ is shown as a positive control. (B) The interaction between SanA and PBP1B was examined using coimmunoprecipitation. Cell lysates containing SanA‐His_6_ and FLAG‐PBP1B^M46‐N844^ were mixed and the anti‐FLAG M2 affinity gel was added to this mixture. The FLAG‐PBP1B^M46‐N844^ was eluted by boiling in SDS‐PAGE loading buffer. The samples were separated using SDS‐PAGE (12%), and western blotting was performed with anti‐FLAG or anti‐SanA antibodies. The blot with anti‐FLAG M2 antibody is shown on the top. The blot with anti‐SanA antibody is shown at the bottom. I: Input; UB: unbound (supernatant of the centrifugation after the mixing of the lysates and M2 agarose); W1–W5: washed samples (supernatant of the centrifugation after washing of M2 agarose); E: eluate (supernatant of the boiled M2 agarose resuspended with 1× SDS‐loading buffer). Three independent experiments were carried out and the typical result is shown.

We next examined whether SanA interacts with PBP1B by the bacterial two‐hybrid (BACTH) assay (Karimova et al. 1998). We could clone *mrcA* encoding PBP1A and *mrcB* encoding PBP1B in the vector pKT25 but not in the vector pUT18C probably because pUT18C is a high copy number plasmid and overproduction of PBP1A or PBP1B could be toxic. SanA showed an interaction signal with PBP1B but not with PBP1A in the BACTH assay (Figure 3A). Because *sanA* ^*M27R*^ was isolated as a suppressor mutation in cells producing RMR, we also examined the interactions between SanA and RMR/RodZ (Figure 3A). SanA did not interact with RMR/RodZ. Only the SanA–PBP1B colonies showed slight blue color. To quantitatively analyze this interaction, we measured the Miller units for the SanA–PBP1A, the SanA–PBP1B interactions, and the negative control. However, none of them showed a significant interaction (control: 139.0 ± 57.8; SanA–PBP1A: 99.4 ± 12.0; SanA–PBP1B: 171.9 ± 95.5, *N* = 6). Thus, the BACTH assay did not provide strong quantitative evidence for a stable interaction between SanA and PBP1B. We therefore decided to biochemically investigate the SanA–PBP1B interaction. The periplasmic fragment of PBP1B^M46‐N844^ was previously used for in vitro assays (Bertsche et al. 2006). Both 6× His‐tagged SanA (SanA‐His_6_) and FLAG‐tagged PBP1B^M46‐N844^ (FLAG‐PBP1B^M46‐N844^) were overexpressed in C41 (DE3) cells. Cell lysates were immunoprecipitated using anti‐His antibodies or anti‐FLAG antibodies. We performed immunoprecipitation multiple times using anti‐His antibodies, but FLAG‐PBP1B^M46‐N844^ was not co‐precipitated with SanA‐His_6_ (Figure S8). It is possible that the amount of SanA‐His_6_ present in the cell was too low relative to FLAG‐PBP1B^M46‐N844^, and thus FLAG‐PBP1B^M46‐N844^ could not be pulled down to a detectable level. Conversely, we performed immunoprecipitation using anti‐FLAG antibodies. SanA‐His_6_ was slightly detected in the elution fraction even in the absence of FLAG‐PBP1B^M46‐N844^, but the amount of SanA‐His_6_ in the elution fraction was clearly increased in the presence of FLAG‐PBP1B^M46‐N844^ (Figure 3B), indicating that SanA and PBP1B can associate under these experimental conditions. Because these experiments were not performed using fully purified proteins, we cannot exclude the possibility that the observed association between SanA and PBP1B is mediated indirectly through one or more intermediary proteins. In the future, if the periplasmic domain of SanA can be purified as a soluble protein, it should be possible to test whether the two purified proteins interact directly in the absence of other factors.

### 2.5 Biological Significance of SanA in *E. coli*

**FIGURE 4:**
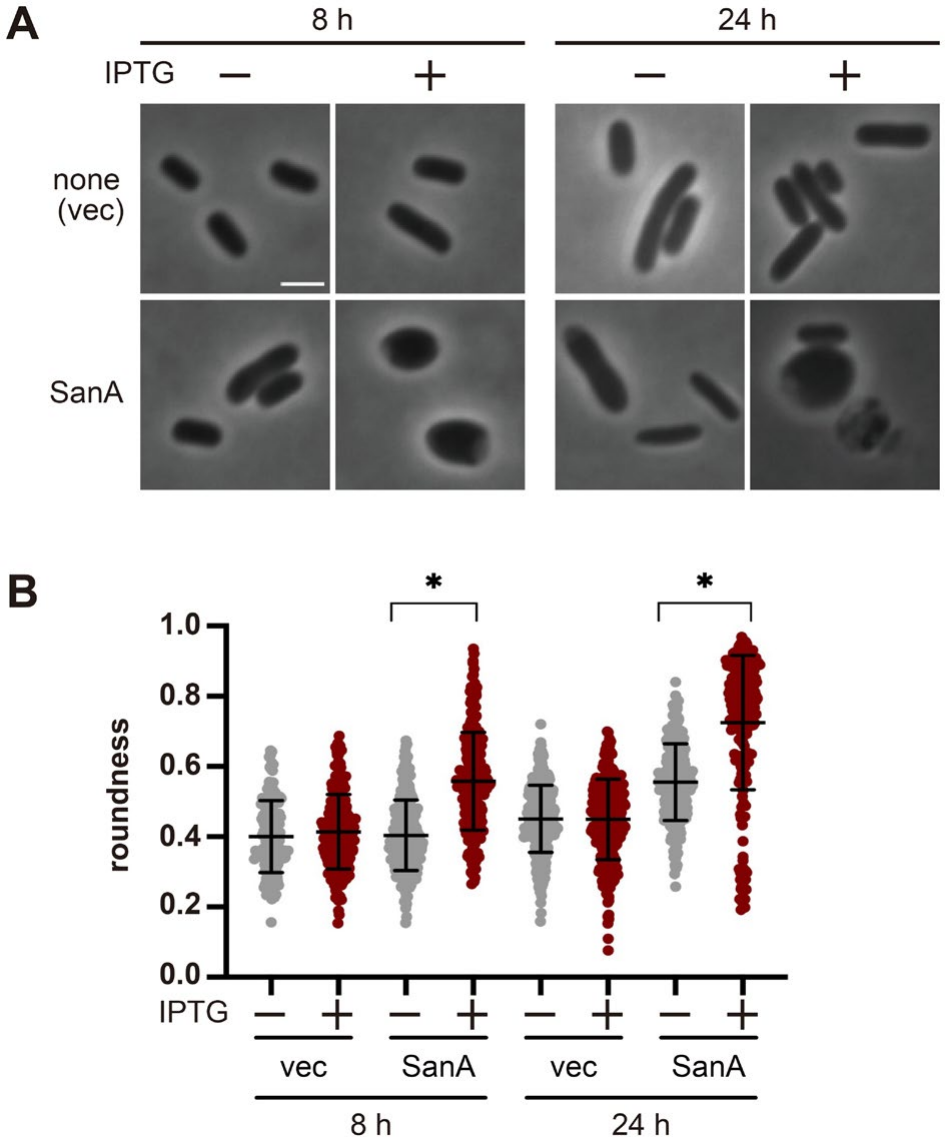
Shape of cells overproducing SanA. WT cells carrying a vector or a plasmid encoding *sanA* were cultured in L medium in the presence or absence of IPTG at 37°C. The cells were observed at the indicated time points. Scale bar: 2 μm. (B) Roundness of each cell at 24 h. The average and standard deviation are shown. *p* values were determined using an unpaired *t*‐test. * shows *p* < 0.0001. [Correction added on 7 March 2026 after first online publication: Figure 4B has been updated.]

**FIGURE 5:**
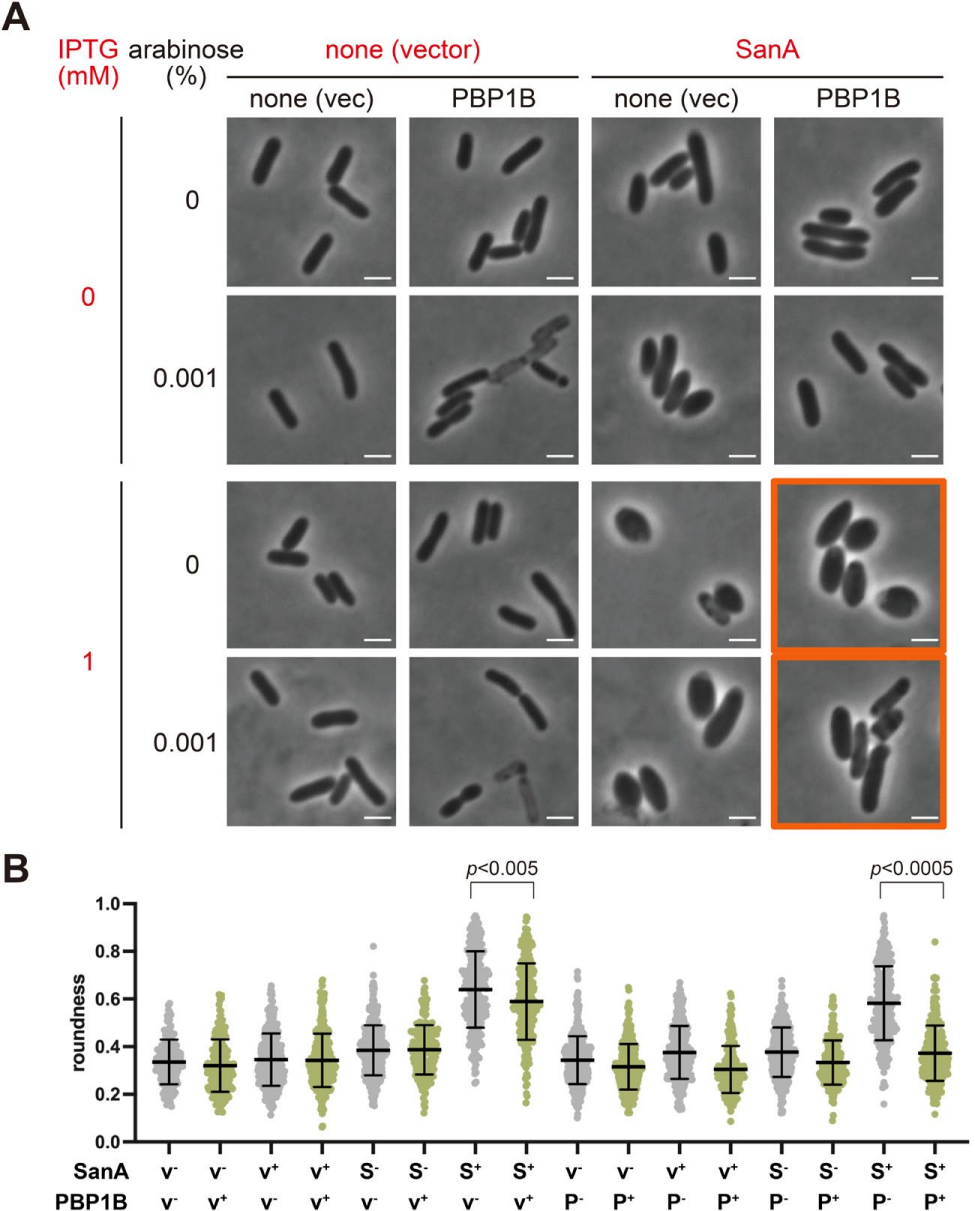
Shape of cells overproducing SanA and PBP1B. (A) WT cells carrying plasmids encoding *sanA*, whose expression was induced with IPTG, and *mrcB*, whose expression was induced with arabinose, were cultured in L medium in the presence or absence of IPTG or arabinose at 37°C for 24 h. Scale bars: 2 μm. (B) Roundness of each cell at 24 h. The average and standard deviation are shown. *p* values were determined using an unpaired *t*‐test. n.s.: *p* > 0.05. v^−^: cells carrying a vector, no inducer; v^+^: cells carrying a vector, with 1 mM IPTG or 0.001% arabinose; S^−^: cells carrying a plasmid encoding *sanA*, no IPTG; S^+^: cells carrying a plasmid encoding *sanA*, with 1 mM IPTG; P^−^: cells carrying a plasmid encoding *mrcB*, no arabinose; S^+^: cells carrying a plasmid encoding *mrcB*, with 0.001% arabinose.

**FIGURE 6:**
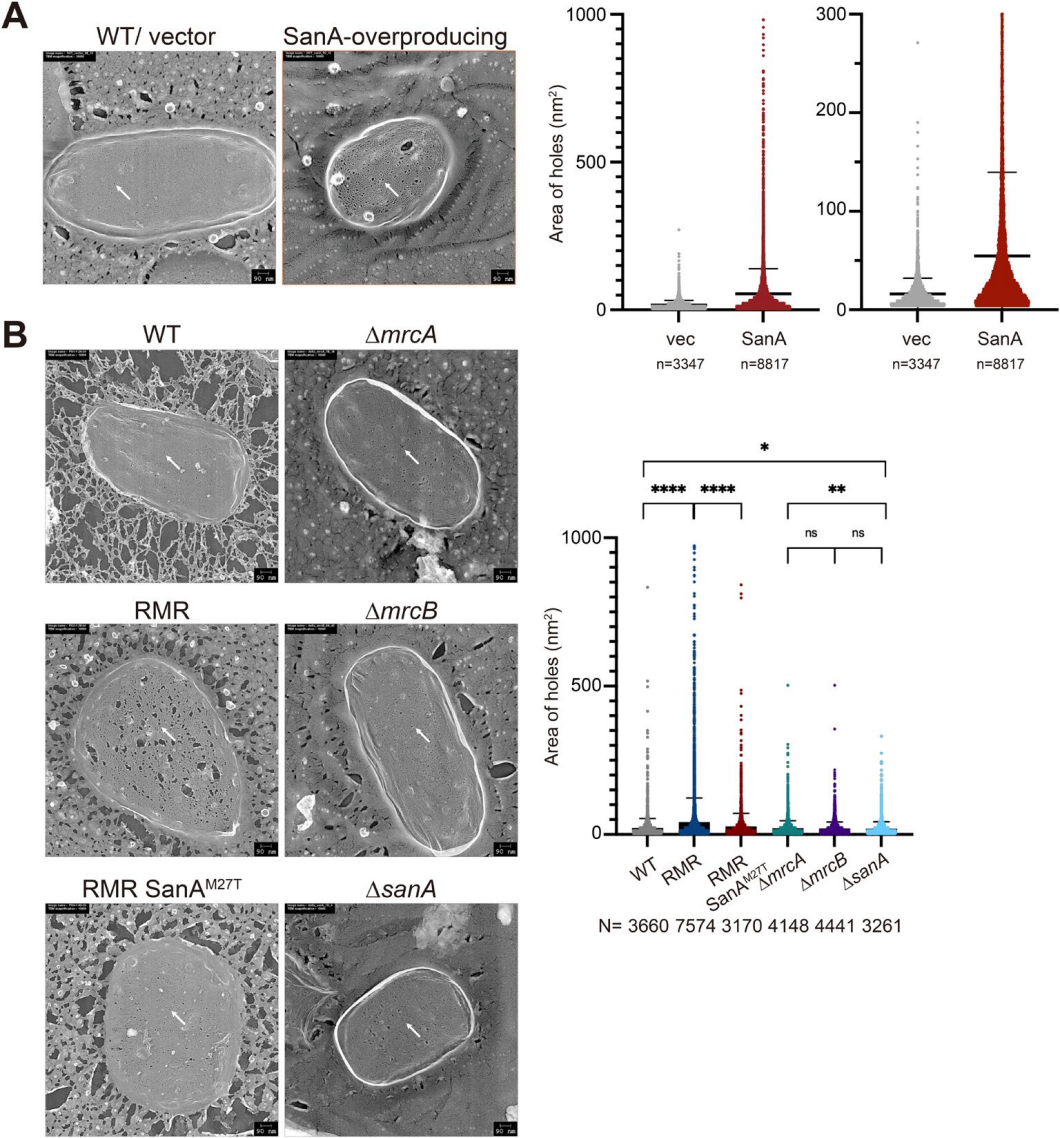
Structure of peptidoglycan. (A) Peptidoglycan purified from cells carrying a vector or a plasmid encoding *sanA* cultured in L medium containing IPTG for 24 h. Peptidoglycan purified from the indicated strain was visualized using quick freeze, deep‐etch, and electron microscopy. Representative pictures are shown. The left graph shows violin plots indicating the distribution of the size of holes in peptidoglycan purified from the indicated strains, whereas the graph on the right enlarges the differences by setting the maximum value of the *y*‐axis to 300 nm^2^. Distribution of the size and number of holes in peptidoglycan purified from each strain. The size and number of holes in the images of the three peptidoglycans were quantified. The average and standard deviation (SD) are shown. The white arrows indicate representative holes. (B) Peptidoglycan purified from RU383 (WT), RU1353 (RMR), RU1696 (RMR SanA^M27R^), RU1998 (∆*mrcA*), RU1999 (∆*mrcB*), or RU1934 (∆*sanA*). The left graph shows violin plots indicating the distribution of the size of holes in peptidoglycan purified from the indicated strains. The white arrows indicate representative holes. *p* values were determined using an unpaired *t*‐test. n.s.: *p* > 0.05, *: 0.01 < *p* ≦ 0.05, **: 0.001 < *p* ≦ 0.01, ****: *p* ≦ 0.0001.

Next, we tried to elucidate the physiological function of SanA in *E. coli* cells. Cells lacking *sanA* displayed a similar morphology and growth rate to WT cells although ∆*sanA* cells were only marginally longer than WT cells as shown above (Figure S6). Then, we examined whether SanA overproduction affected the cell shape. To overproduce SanA, we cloned the *sanA* gene in an IPTG‐inducible plasmid. Expression of *sanA* from this plasmid resulted in approximately a 50‐fold increase in the absence of IPTG and up to a 1500‐fold increase in the presence of IPTG, compared with the level expressed from the chromosome (Figure S9). Overproduction of SanA in WT cells changed the cell shape from rod to round, with some round cells being lysed at the cell poles (Figure 4A, WT). We measured the roundness of cells (Figure 4B). Although the roundness of cells carrying the plasmid encoding *sanA* increased even without IPTG, the roundness significantly increased in the presence of IPTG probably because of the weakening of peptidoglycan due to the overproduction of SanA, which causes the cells to become spherical because they cannot withstand turgor pressure. If so, the cells do not become spherical in a hyperosmotic medium (NBMSM), even when SanA is overproduced. In fact, the cells remained rod‐shaped when SanA was overproduced in NBMSM (Figure S10), supporting the idea that the peptidoglycan in cells overproducing SanA in the L medium was weakened (see also Figure 6). Because SanA is associated with PBP1B (Figure 3) and if the cell rounding by overproduction of SanA is somehow caused by the interaction between SanA and PBP1B, the overproduction of PBP1B should suppress cell rounding caused by the overproduction of SanA. We cloned *mrcB* (encoding PBP1B) in an arabinose‐inducible plasmid. Arabinose addition slightly suppressed cell rounding caused by the overproduction of SanA with 1 mM IPTG in cells carrying an empty vector for *mrcB* (Figure 5A,B). However, cells carrying the plasmids encoding *sanA* and *mrcB* became spherical or ovoid when grown in the presence of 1 mM IPTG; in contrast, when these cells were cultured in the presence of both 1 mM IPTG and 0.001% arabinose, they reverted to a rod‐shaped morphology, suggesting that the suppression of cell rounding due to the overproduction of SanA by PBP1B was more effective than the suppression caused by the addition of arabinose (Figure 5A,B). To assess the amount of PBP1B protein under these conditions, we constructed a plasmid expressing PBP1B‐3 × FLAG, in which a 3 × FLAG tag was fused to the C‐terminus of PBP1B (an identical plasmid to the plasmid expressing untagged PBP1B except for the presence of the FLAG tag), as well as a strain expressing PBP1B‐3 × FLAG from the chromosome. First, to determine whether PBP1B‐3 × FLAG is functional, we introduced the ∆*mrcA::kan* allele into the strain via P1 transduction. As controls, the same experiment was performed using a WT strain and a ∆*mrcB* mutant. Consistent with previous reports, ∆*mrcA::kan* could not be introduced into the ∆*mrcB* mutant, whereas it could be successfully introduced into both WT and PBP1B‐3 × FLAG strains (Figure S11A). These results indicate that PBP1B‐3 × FLAG retains its function as PBP1B. Next, to examine the degree of overproduction when PBP1B‐3 × FLAG was expressed from the plasmid, we performed immunoblotting using an anti‐FLAG antibody. As a result, the expression level of PBP1B‐3xFLAG in the absence of arabinose was comparable to that of chromosomally expressed PBP1B‐3 × FLAG (~1.8‐fold), whereas induction with arabinose resulted in approximately a 2.2‐fold increase in expression in cells grown to log‐phase (Figure S11B, top). In cells grown to stationary phase, expression level of PBP1B and PBP1B‐3xFLAG from this plasmid resulted in approximately a 20‐fold increase in the absence of arabinose, whereas induction with arabinose resulted in approximately a 250‐fold increase (Figure S11B, bottom). We also examined whether the overproduction of LpoB, an activator of PBP1B, suppressed cell rounding caused by SanA overproduction. Because LpoB positively regulates PBP1B activity, we tested whether the phenotype caused by SanA overproduction could be counteracted by increased LpoB levels. However, unlike PBP1B, the overproduction of LpoB did not suppress cell rounding caused by SanA overproduction (Figure S12). These observations suggest that the cell rounding caused by SanA overproduction is associated with PBP1B‐dependent processes, as the phenotype is suppressed by increased PBP1B levels but not by LpoB overproduction and that the rounded morphology likely reflects weakened peptidoglycan that is unable to withstand turgor pressure.

### 2.6 Structure of Peptidoglycan Upon SanA Overproduction

If SanA overproduction affects peptidoglycan synthesis, the structure of peptidoglycan produced under these conditions would be expected to be altered. We recently employed quick‐freeze, deep‐etch electron microscopy (QFDE‐EM) to visualize peptidoglycans purified from WT, RMR, and RMR suppressors (except for SanA^M27R^) (Ago et al. 2023). Here, we purified peptidoglycans from WT cells carrying an empty vector or a plasmid encoding the *sanA* gene grown in the presence of IPTG and observed them using QFDE‐EM (Figure 6A). The peptidoglycan purified from cells overproducing SanA had larger holes, and the number of holes was higher than that in the peptidoglycan purified from cells carrying an empty vector, consistent with the idea that the peptidoglycans in cells overproducing SanA are weaker than those of WT cells, as expected from previous results (Figure 4, Figure 5, Figure S10).

### 2.7 Mechanism of Suppression of RMR by the SanA Mutant

Thus far, our data suggest that SanA influences peptidoglycan synthesis through PBP1B‐dependent processes. We previously showed that the peptidoglycan of RMR had larger holes, and the number of holes was higher than that in the WT (Figure 6B, Table 2) (Ago et al. 2023). The number and size of holes in the peptidoglycan of RMR SanA^M27R^ cells were clearly reduced compared with those of RMR cells (Figure 6B, Table 2), indicating that SanA^M27R^ suppressed the defects in the peptidoglycan of RMR cells. Because *sanA* ^*M27R*^ is a loss‐of‐function mutation, these results suggest that peptidoglycan defects in RMR cells are no longer constrained by SanA, allowing PBP1B‐dependent peptidoglycan synthesis to contribute more effectively to cell wall maintenance. Consistent with this interpretation, overproduction of PBP1B slightly rescued the slow‐growth phenotype of RMR cells while the cell shape was not affected (Figure S13). Our data suggest that two mechanisms are involved in suppressing the defect of peptidoglycan in RMR cells: (i) the stimulation of peptidoglycan synthesis activity of the Rod complex by a mutation, such as MreB^A125V^ or PBP2^T52I^ (Ago et al. 2023), and (ii) increased contribution of PBP1B‐dependent peptidoglycan synthesis, as investigated in this study. This interpretation implies that not only the Rod complex but also PBP1B contributes to the peptidoglycan synthesis required for cell elongation. Consistent with this idea, the ∆*mrcB*::*kan* allele could not be introduced into RMR cells, whereas ∆*mrcA*::*kan* was readily transduced into both WT and RMR cells (Figure S14A), suggesting that PBP1B becomes essential under conditions of reduced Rod‐complex activity. Furthermore, when SanA was overexpressed in cells producing MreB^A125V^ or PBP2^T52I^ which are assumed to have a higher peptidoglycan synthesis activity, the cells became thinner but not spherical (Figure S14B). These results indicate that a proper balance between the activities of the Rod complex and PBP1B is critical for maintaining peptidoglycan integrity and cell viability.

**TABLE 2:** The number and size of holes in purified peptidoglycan.

Peptidoglycan purified from	Number^a^	Size (nm^2^)^b^
RU383 (WT)^c^	3660	21.7 ± 31.7
RU1353 (RMR)^c^	7574	42.1 ± 81.1
RU1696 (RMR SanA^M27R^)	3170	27.1 ± 44.2
RU1998 (∆*mrcA*)	4118	21.5 ± 25.2
RU1999 (∆*mrcB*)	4441	20.6 ± 21.8
RU1934 (∆*sanA*)	3261	20.0 ± 22.9

^a^ Sum of the holes of three purified peptidoglycan surfaces.

^b^ Mean ± standard deviation (nm^2^) of the size of the holes of three purified peptidoglycan surfaces.

^c^ Data sets of RU383 (WT) and RU1353 (RMR) were quoted from our previous paper (Ago et al. 2023).

### 2.8 Structure of Peptidoglycan of Cells Lacking *mrcA* , *mrcB* , or *sanA*

As discussed above, if SanA influences peptidoglycan synthesis through PBP1B‐dependent processes, structural differences in peptidoglycan would be expected in the absence of *sanA*. To test this possibility, we purified peptidoglycan from ∆*sanA*, ∆*mrcA*, and ∆*mrcB* cells and analyzed their structures as described above (Figure 6B, Table 2). The holes in peptidoglycan purified from ∆*sanA* cells were slightly—but statistically significantly—smaller than those in peptidoglycan from WT and ∆*mrcA* (PBP1B^+^) cells. In contrast, the hole sizes of peptidoglycan purified from the ∆*sanA* strain showed no statistically significant difference compared with those from the ∆*mrcB* strain. These results indicate that the effect of SanA on peptidoglycan structure is observed only in cells in which PBP1B is present, suggesting that SanA influences peptidoglycan architecture in a PBP1B‐dependent manner. Collectively, these findings support the notion that SanA affects peptidoglycan synthesis and/or repair through processes that require PBP1B.

## 3 Conclusions

Suppressor mutants of cells functionally deficient in the Rod complex have been obtained in several studies (Shiomi et al. 2013; Shiomi and Niki 2013; Morgenstein et al. 2015; Rohs et al. 2018; Ago et al. 2023) except for a mutation in the promoter region of *zipA* (Shiomi and Niki 2013). This is expected, as the functional defect in the Rod complex is suppressed by mutations in other factors within the Rod complex, either by enhancing the activity of the complex itself or by interaction between the components. However, for the first time, a mutation in SanA, a factor distinct from the known Rod complex factors, was identified as a suppressor mutation in the RMR and ∆*rodZ* cells. We identified SanA as a novel factor associated with PBP1B. SanA may influence peptidoglycan synthesis and/or repair through PBP1B‐dependent processes. Thus, PBP1B function is positively and negatively regulated by LpoB and SanA, respectively. LpoB interacts with the UB2H domain of PBP1B (Paradis‐Bleau et al. 2010; Typas et al. 2010; Egan et al. 2014; King et al. 2014). Our data (Figure S12) suggest that SanA does not compete with LpoB for binding to PBP1B.

During the preparation of this manuscript, one paper on SanA was published (Gundavarapu et al. 2025) and another preprint was made available (Carr et al. 2025). In the former study, it was proposed that SanA negatively regulates peptidoglycan synthesis. The authors reported that peptidoglycan synthesis was increased in the absence of SanA, without major changes in its chemical composition—for example, in the ratio of 4–3 to 3–3 crosslinks. In the latter study, it was demonstrated that loss of *sanA* results in pronounced envelope permeability defects under conditions of elevated Lipid II availability. These defects could be suppressed by delaying septal peptidoglycan synthesis through mutations in *ftsI*. These findings suggest that SanA functions to limit the excessive supply and utilization of Lipid II. In the present study, we showed that SanA physically interacts with PBP1B and may negatively regulate its activity. Since PBP1B uses Lipid II as its substrate, the activity level of PBP1B is a key determinant of Lipid II consumption. Thus, it is likely that SanA regulates the availability of Lipid II by modulating PBP1B activity. In the absence of SanA, increased PBP1B‐dependent synthesis may consume larger amounts of Lipid II, thereby promoting increased peptidoglycan synthesis and/or repair. We observed that overproduction of SanA occasionally causes the poles of oval‐shaped cells to appear eroded or partially lysed. Because cell poles are formed during or after cell division, this observation raises the possibility that SanA is associated with proteins localized at the division site or cell poles. Notably, two other studies have reported roles of SanA in cell division, which is consistent with this idea. These findings suggest that SanA may interact not only with PBP1B but also with other proteins and could function as a multifunctional regulator of peptidoglycan synthesis. Thus, the findings from these three independent studies and preprints can be reconciled to support a coherent model of SanA function.

## 4 Materials and Methods

### 4.1 Bacterial Strains and Growth Conditions

All strains were derivatives of *E. coli* K‐12 and are listed in Table S2. BW25113 is a WT strain (Baba et al. 2006), and RU383 (sfGFP‐RodZ) and RU1353 (sfGFP‐RMR) produce RodZ fused with sfGFP (Ikebe et al. 2018) and RMR, a chimeric protein in which the TM domain of RodZ was replaced with the first TM domain of MalF, fused with sfGFP (Ago et al. 2023), respectively. Cells were grown in L broth (1% Bacto tryptone, 0.5% yeast extract, 0.5% NaCl) at 37°C. Kanamycin (Kan; 50 μg/mL), ampicillin (Amp; 100 μg/mL), and chloramphenicol (Cm; 20 μg/mL) were added to the culture medium when necessary. The absorbance (OD_660_) was measured every 5 min using a compact rocking incubator (TVS062CA; ADVANTEC).

### 4.2 Strain Constructions

The primers used for the construction of strains are listed in Table S3.

Construction of RU1651 (Δ*cdd*::cat), RU1652 (*sfGFP‐rmr sanA* ^*M27R*^ ∆*cdd*::cat), RU1695 (*sfgfp‐rodZ* Δ*cdd::cat sanA* ^*M27R*^), RU1696 (*sfgfp‐rmr* Δ*cdd::cat sanA* ^*M27R*^), RU1724 (Δ*cdd::cat sanA* ^*M27R*^), RU1861 (∆*sanA*::kan), RU1934 (∆*sanA*), RU1865 (Δ*cdd::cat* Δ*sanA::kan*), and RU1949 (*sfgfp‐rmr* Δ*cdd::cat* ∆*sanA*::kan): For transducing the *sanA* ^*M27R*^ mutation, we first inserted the chloramphenicol acetyltransferase (*cat*) gene into *cdd*, which is located adjacent to *sanA* on the genome. Polymerase chain reaction (PCR) was performed using pKD3 as the template and the primers 1895/1896. The PCR product was introduced into BW25113 (WT) and RU1478 (original suppressor of RU1353 carrying *sanA* ^*M27R*^) to yield RU1651 (Δ*cdd*::cat) and RU1652 (*sfGFP‐rmr sanA* ^*M27R*^ ∆*cdd*::cat). P1 phage was grown on RU1652 and used for transducing RU383 (sfGFP‐*rodZ*), RU1353 (sfGFP‐rmr), and BW25113 (WT). Fresh transductants were restreaked on L plates containing Cm, and Cm^R^ clones were selected to obtain RU1695 (*sfgfp‐rodZ* Δ*cdd::cat sanA* ^*M27R*^), RU1696 (*sfgfp‐rmr* Δ*cdd::cat sanA* ^*M27R*^), and RU1724 (Δ*cdd::cat sanA* ^*M27R*^). P1 phage was grown on JW2132 (∆*sanA*::kan) and then used for transducing BW25113 (WT). Fresh transductants were restreaked on L plates containing Kan, and Kan^R^ clones were selected to obtain RU1861 (∆*sanA*::kan). RU1861 was transformed with pCP20 using selection for Amp resistance (Amp^R^) at 30°C. The strain was then incubated at 42°C in the absence of Amp, and the colonies that grew were screened for Amp‐ and Kan‐sensitive phenotype at 37°C. The resulting strain was designated RU1934 (∆*sanA*). ∆*sanA*::kan was amplified using JW2132 (∆*sanA*::kan) and the primers 1865/1866. The PCR product was introduced into RU1651 to obtain RU1865 (Δ*cdd::cat* Δ*sanA::kan*). P1 phage was grown on RU1865 (Δ*cdd::cat* Δ*sanA::kan*) and used for transducing RU1353 (sfGFP‐*rmr*). Fresh transductants were restreaked on L plates containing Cm and Kan, and Cm^R^ Kan^R^ clones were selected to obtain RU1949 (sfGFP‐*rmr* Δ*cdd::cat* ∆*sanA*::kan). RU1865 (Δ*cdd::cat* Δ*sanA::kan*) was transformed with plasmid pCP20 by selection for ampicillin resistance (Amp^R^) at 30°C. The strain was then incubated at 42°C in the absence of Amp, and colonies that grew were screened for Amp^S^ and Cm^S^ phenotypes at 37°C. The resulting strain was designated as RU1933 (Δ*cdd* Δ*sanA*).

Construction of RU1635 (∆*rodZ::kan*), RU1636 (*sanA* ^*M27R*^ ∆*rodZ::kan*), and RU2994 (*∆sanA* Δ*rodZ::kan*): P1 phage was grown on RU2 (Δ*rodZ::kan*). The P1 phages were used for transducing RU1651 (∆*cdd::cat*), RU1724 (∆*cdd::cat sanA* ^*M27R*^), and RU1933 (Δ*cdd* Δ*sanA*). Fresh transductants were restreaked on L plates containing Kan, and Kan^R^ clones were selected to obtain RU1635 (∆*rodZ::kan*), RU1636 (*sanA* ^*M27R*^ ∆*rodZ::kan*), and RU2994 (*∆sanA* Δ*rodZ::kan*).

Construction of RU1965 (∆*mrcA*::*kan*), RU1998 (∆*mrcA*), RU1966 (∆*mrcB*::*kan*), RU1999 (∆*mrcB*), RU2091 (∆*mrcA* ∆*sanA*::*kan*), RU2110 (∆*mrcA* ∆*sanA*), RU2092 (∆*mrcB* ∆*sanA*::*kan*), RU2112 (∆*mrcB* ∆*sanA*), RU2203 (∆*lpoA*::*kan*), RU2208 (∆*lpoA*), RU2204 (∆*lpoB*::*kan*), RU2209 (∆*lpoB*), RU2205 (∆*sanA* ∆*lpoA*::*kan*), RU2210 (∆*sanA* ∆*lpoA*), RU2206 (∆*sanA* ∆*lpoB*::kan), and RU2211 (∆*sanA* ∆*lpoB*): P1 phage was grown on JW3359 (∆*mrcA*::kan), JW0145 (∆*mrcB*::kan), JW3116 (∆*lpoA*::kan), and JW5157 (∆*lpoB*::kan). The P1 phages were used for transducing BW25113 (WT) or RU1934 (∆*sanA*). Fresh transductants were restreaked on L plates containing Kan, and Kan^R^ clones were selected to obtain RU1965 (∆*mrcA*::*kan*), RU1966 (∆*mrcB*::*kan*), RU2203 (∆*lpoA*::*kan*), RU2204 (∆*lpoB*::*kan*), RU2205 (∆*sanA* ∆*lpoA*::*kan*), and RU2206 (∆*sanA* ∆*lpoB*::*kan*), respectively. These strains were transformed with pCP20 using selection for Amp resistance (Amp^R^) at 30°C. The strains were then incubated at 42°C in the absence of Amp, and the colonies that grew were screened for Amp‐ and Kan‐sensitive phenotype at 37°C. The resulting strains were designated RU1998 (∆*mrcA*), RU1999 (∆*mrcB*), RU2208 (∆*lpoA*), RU2209 (∆*lpoB*), RU2210 (∆*sanA* ∆*lpoA*), and RU2211 (∆*sanA* ∆*lpoB*). The P1 phage (∆*sanA*::*kan*) was used for transducing RU1998 (∆*mrcA*) and RU1999 (∆*mrcB*). Fresh transductants were restreaked on L plates containing Kan, and Kan^R^ clones were selected to obtain RU2091 (∆*mrcA* ∆*sanA*::*kan*) and RU2092 (∆*mrcB* ∆*sanA*::*kan*). These strains were transformed with pCP20 using selection for Amp resistance (Amp^R^) at 30°C. The strains were then incubated at 42°C in the absence of Amp, and the colonies that grew were screened for Amp‐ and Kan‐sensitive phenotype at 37°C. The resulting strains were designated RU2110 (∆*mrcA* ∆*sanA*) and RU2112 (∆*mrcB* ∆*sanA*).

Construction of RU2979 (*mrcB‐3xflag*, Cm^R^) and RU2993 (*mrcB‐3xflag*, Cm^S^): DNA fragment containing 3xflag, Cm^R^ gene sandwiched by FRT sequences was amplified by PCR with genomic DNA of SN208 (MG1655 *panD‐3xflag*) (Nozaki and Niki 2012) as a template and primers 3223/3224. The PCR product was introduced into strain BW25113 carrying pKD46 (Datsenko and Wanner 2000) by electroporation. Cells were selected on L plates containing 10 μg mL^−1^ Cm. The resulting strain (RU2979) was transformed with plasmid pCP20 by selection for ampicillin resistance (Amp^R^) at 30°C. The strain was then incubated at 42°C in the absence of Amp, and colonies that grew were screened for Amp^S^ and Cm^S^ phenotypes at 37°C. The resulting strain was designated as RU2993.

Construction of RU2879 (MG1655 *∆sanA::kan*) and RU2880 (MG1655 *∆eylC::kan*): P1 phage was grown on RU1861 (Δ*sanA::kan*) and JW0903 (Δ*eylC::kan*). The P1 phages were used for transducing MG1655 (WT). Fresh transductants were restreaked on L plates containing Kan, and Kan^R^ clones were selected to obtain RU2879 (MG1655 *∆sanA::kan*) and RU2880 (MG1655 *∆eylC::kan*).

### 4.3 Plasmid Construction

The primers and plasmids used in this study are listed in Tables S3 and S4, respectively. PCR was performed using DNA polymerase KOD One (Toyobo, Japan).

For constructing the plasmids used in the BACTH assay, *sanA*, *mrcA*, and *mrcB* were amplified using BW25113 as a template and the primers 1874/1875, 2145/2146, and 2147/2148 for *sanA*, *mrcA*, and *mrcB*, respectively. The PCR products were digested with BamHI and EcoRI, and the fragments were cloned into the corresponding sites in pKT25 and pUT18C to obtain pRU1697 (pKT25‐*sanA*), pRU1623 (pUT18C‐*sanA*), pRU2105 (pKT25‐*mrcA*), pRU2106 (pKT25‐*mrcB*), and pRU2340 (pUT18C‐*mrcB*). Each point mutant was introduced into pRU1697 (pKT25‐*sanA*), pRU1623 (pUT18C‐*sanA*), pRU2106 (pKT25‐*mrcB*), and pRU2340 (pUT18C‐*mrcB*) via two‐step site‐directed mutagenesis using the primers listed in Table S3. Some of the mutant plasmids were constructed using the In‐Fusion system (Takara, Japan). We confirmed that the plasmids created using either method had the same sequence, except for the mutation site.

For constructing a plasmid carrying *sanA*, we first constructed pRU1565 (pDSW208‐∆*gfp*). The PstI‐ScaI fragment containing *gfp* of pDSW208 was replaced with the corresponding fragment, which did not contain *gfp* of pDSW207, to obtain pRU1565 (pDSW208‐∆*gfp*). *sanA* gene was amplified using BW25113 as a template and the primers 1872 and 1873. The PCR product was digested with EcoRI and HindIII and the fragment was cloned into the corresponding sites in pRU1565 to obtain pRU1582 (pDSW208‐∆*gfp*‐*sanA*). To construct pRU1964 (pTrc99A‐kan‐*sanA*), PCR was carried out using pRU1582 (pDSW208‐∆*gfp*‐*sanA*) as a template and primers 2064/2093. The PCR product was digested with NcoI and XbaI and the fragment was cloned into the corresponding site in pRU1825 (pTrc99A‐kan) to obtain pRU1964 (pTrc99A‐kan‐*sanA*).

For constructing the plasmid used for SanA‐His_6_ overproduction, the *sanA* gene was amplified using BW25113 as a template and the primers 1926 and 1927. The PCR products were digested with NcoI and XhoI, and the fragments were cloned into the corresponding sites in pET28Ap to obtain pRU1693 (pET28Ap‐*sanA*‐*his*_*6*_). For constructing the plasmid carrying FLAG (DYKDDDDK)‐VKLLE‐*mrcB* ^*M46‐N844*^, *mrcB* ^*M46‐N844*^ was amplified using BW25113 and pET28Ap as templates and the primers 2394/2396 and 2396/2397, respectively. The PCR products were ligated using the In‐Fusion system to obtain pRU2417 (pET28Ap‐FLAG (DYKDDDDK)‐VKLLE‐*mrcB* ^*M46‐N844*^). For constructing the control plasmid (pET28Ap‐FLAG), PCR was carried out using pRU2417 as a template and the primers 2514/2515. The PCR products were ligated using the iVec method (Nozaki and Niki 2019). However, the resulting plasmid contained a duplicated KpnI site. Therefore, the plasmid was digested with KpnI, and the digested fragment was ligated to obtain pRU2502 (pET28Ap‐FLAG). For constructing the plasmid for SanA‐His_6_ and FLAG‐PBP1B^M46‐N844^, PCR was carried out using pET‐duet and pRU1693 (pET28Ap‐*sanA*‐*his*_*6*_) as templates and the primers 3227/3228 and 3225/3226, respectively. The PCR products were ligated using the In‐Fusion system (Takara‐bio, Shiga, Japan) to obtain pRU2980 (pET‐duet‐*sanA*‐*his*_*6*_). PCR was carried out using pET‐duet and pRU2417 (pET28Ap‐FLAG‐*mrcB* ^*M46‐N844*^) as templates and the primers 3231/3232 and 3229/3230, respectively. The PCR products were ligated using the In‐Fusion system to obtain pRU2981 (pET‐duet‐FLAG‐*mrcB* ^*M46‐N844*^). PCR was carried out using pRU2980 (pET‐duet‐*sanA*‐*his*_*6*_) and pRU2417 (pET28Ap‐FLAG‐*mrcB* ^*M46‐N844*^) as templates and the primers 3231/3232 and 3229/3230, respectively. The PCR products were ligated using the In‐Fusion system to obtain pRU2982 (pET‐duet‐*sanA*‐*his*_*6*_/FLAG‐*mrcB* ^*M46‐N844*^).

For constructing the arabinose‐inducible plasmid carrying *mrcB*, we first constructed pRU1276 (pBAD33‐MCS3). The ClaI‐HindIII fragment containing multiple cloning site 2 (MCS2) of pBAD33 was replaced with the corresponding fragment containing MCS3 of pBAD24 to obtain pRU1276 (pBAD33‐MCS3). PCR was performed using BW25113 and pRU1276 as templates and the primers 2390/2392 and 2391/2393. The PCR products were ligated using the In‐Fusion system to obtain pRU2416 (pBAD33‐MCS3‐*mrcB*). For constructing the arabinose‐inducible plasmid carrying *mrcB*‐*3× flag*, PCR was carried out using RU2993 (BW25113 *mrcB‐3× flag*) and pRU1276 (pBAD33‐MCS3) as templates and the primers 3243/3244 and 2392/3245, respectively. The PCR products were ligated using the In‐Fusion system to obtain pRU2998 (pBAD33‐MCS3‐*mrcB‐3xflag*).

### 4.4 Isolation of Suppressors of the Slow Growth Phenotype of RMR Cells

Suppressors of the slow‐growth phenotype of RU1353 (sfGFP‐RMR) were isolated and sequenced as described (Ago et al. 2023).

### 4.5 Microscopic Observations and Image Analyses

Cells were grown in L medium at 37°C and mounted on 2% agarose in M9 medium (0.6% Na_2_HPO_4_, 0.3% K_2_HPO_4_, 0.05% NaCl, 0.1% NH_4_Cl, 0.1 mM MgSO_4_·7H_2_O, 0.2% glucose) (M9‐agarose pad). Cells were observed using an Axio Observer (Zeiss), and images were processed using ZEN (Zeiss), Photoshop (Adobe), Affinity Photo2 (Serif), and ImageJ. All experiments were repeated two or more times on different days. Cells were detected and counted automatically using ImageJ and the MicrobeJ plug‐in (Ducret et al. 2016). All cells in the images, except overlapping cells, were counted. The size of the Rod complex (sfGFP‐RodZ or sfGFP‐RMR foci) was quantified as previously described (Ago et al. 2023).

### 4.6 Prediction of Three‐Dimensional Structures of SanA and PBP1B

Three‐dimensional structures of SanA were predicted using AlphaFold2 (https://colab.research.google.com/github/sokrypton/ColabFold/blob/main/AlphaFold2.ipynb). The structures were visualized using UCSF Chimera (https://www.cgl.ucsf.edu/chimera/).

### 4.7 Bacterial Two‐Hybrid Assay

The pKT25 and pUT18C plasmids carrying *sanA*, *mrcA* (encoding PBP1A), or *mrcB* (encoding PBP1B) genes were used to transform DHM1 cells (∆*cya*). Bacterial two‐hybrid assays were performed as previously described (Shiomi and Margolin 2007; Yoshii et al. 2019; Ago et al. 2023).

### 4.8 Coimmunoprecipitation With Ni‐NTA or Anti‐FLAG M2

Overnight cultures of C41 (DE3) carrying pRU2980 (pET‐duet‐*sanA‐his*_*6*_), pRU2981 (pET‐duet‐ *flag*‐*mrcB* ^*M46‐N844*^), or pRU2982 (pET‐duet‐*sanA‐his*_*6*_/ *flag*‐*mrcB* ^*M46‐N844*^) were diluted 100‐fold in 40 mL of L medium containing Amp and grown to the log phase (OD_600_ = 0.5–0.6) at 37°C. Then, 1 mM IPTG was added, and the cultures were grown for 2 h at 37°C. Cells were centrifuged at 10,000×*g* for 10 min at 4°C to collect the bacteria. The cells were suspended in 4 mL of buffer A (100 mM Tris–HCl [pH 7.5], 150 mM NaCl, and 2% [w/v] sodium cholate) and stirred at room temperature for 15 min. Thereafter, the sample was sonicated and centrifuged at 16,000×*g* for 20 min at 4°C.

500 μL of Ni‐NTA resin (Qiagen) was mixed with 1 mL of cell lysate and the mixture was incubated for 1 h at 4°C. Then, the sample was centrifuged at 1000×*g* for 1 min at 4°C. The resin was washed with 1 mL of buffer A containing the indicated concentration of imidazole.

40 μL of Anti‐FLAG M2 Affinity Gel (Sigma‐Aldrich) was mixed with 1 mL of cell lysate and the mixture was incubated for 1 h at 4°C. The sample was centrifuged at 8000×*g* for 1 min at 4°C, and the affinity resin was resuspended in 1 mL of buffer A. The samples were then washed three times. Thereafter, 100 μL of 1× SDS‐loading buffer was added to the resin and the sample was incubated for 5 min at 95°C.

### 4.9 Immunoblotting

The samples were separated using SDS‐PAGE (12%), and immunoblotting was performed using anti‐SanA (Eurofins) or anti‐FLAG (Sigma‐Aldrich) antibodies as primary antibodies, and horseradish peroxidase (HRP)‐conjugated anti‐rabbit IgG antibody (Jackson ImmunoResearch) or HRP‐conjugated anti‐mouse IgG antibody (Jackson ImmunoResearch) as secondary antibodies. The signal was detected on an Amersham ImageQuant 800 (Cytiva) using ECL Select Western Blotting Detection Reagent (Cytiva). The bands were quantified using the ImageJ software (https://imagej.nih.gov).

### 4.10 Visualization of Peptidoglycan Using QFDE‐EM

Peptidoglycan purification and visualization using QFDE‐EM were performed as described previously (Tulum et al. 2019; Ago et al. 2023). Holes smaller than 4 nm^2^ and larger than 1000 nm^2^ were excluded from quantification.

### 4.11 Statistics and Reproducibility

Statistical analysis was performed with GraphPad Prizm 10.5.0. The mean and SD of the data were used to create the graphs, and *p*‐values were obtained by unpaired *t*‐tests. *p*‐value < 0.05 was defined as a significant difference. All the experiments were repeatedly carried out, and representative results are shown.

## Author Contributions

**Honoka Yamaguchi:** conceptualization; methodology; data curation; investigation; validation; formal analysis; visualization; writing – review and editing. **Risa Ago:** conceptualization; investigation. **Yuhei O. Tahara:** methodology; data curation; investigation; validation; formal analysis; visualization. **Mari Inoue:** investigation; validation; formal analysis; visualization. **Hironori Niki:** conceptualization; supervision; funding acquisition. **Makoto Miyata:** conceptualization; supervision; funding acquisition. **Daisuke Shiomi:** conceptualization; methodology; data curation; investigation; validation; formal analysis; supervision; funding acquisition; visualization; project administration; writing‐original draft; writing – review and editing.

## Funding

This work was supported by the Japan Science and Technology Agency (JST) CREST, JPMJCR19S5. NIG‐JOINT, 57A2018, 60A2019, 59A2021, 47A2022, 64A2023, 55A2024, 32A2025.

## Ethics Statement

The authors have nothing to report.

## Conflicts of Interest

The authors declare no conflicts of interest.

## Supporting information


**Data S1:** mmi70058‐sup‐0001‐supinfo.pdf.

## Data Availability

The data that support the findings of this study are available from the corresponding author upon reasonable request.
