# Supplementary material for: SanA Plays a Role in Peptidoglycan Integrity in Escherichia coli
Source: Mol Microbiol. 2026 Feb 20;125(4):343–57. doi: 10.1111/mmi.70058 (PMC13046291; doi:10.1111/mmi.70058)
Supplement: Supplementary file 1 — Data S1: mmi70058‐sup‐0001‐supinfo.pdf. [file MMI-125-343-s001.pdf]

SanA plays a role in peptidoglycan integrity in *Escherichia coli*

Honoka Yamaguchi[1], Risa Ago[1], Yohei O Tahara[2,3], Mari Inoue[1], Hironori Niki[4,5], Makoto Miyata[2,3], Daisuke Shiomi[1]*

[1]Department of Life Science, College of Science, Rikkyo University
[2] Graduate School of Science, Osaka Metropolitan University
[3] The OMU Advanced Research Center for Natural Science and Technology, Osaka Metropolitan University
[4]Microbial Physiology Laboratory, Department of Gene Function and Phenomics, National Institute of Genetics, Japan
[5]Department of Genetics, The Graduate University for Advanced Studies, SOKENDAI

*** Correspondence:**
Daisuke Shiomi: dshiomi@rikkyo.ac.jp, Department of Life Science, College of Science, Rikkyo University, 3-34-1 Nishi Ikebukuro, Toshima-ku, Tokyo 171-8501, Japan

Keywords: peptidoglycan, Rod complex, *E. coli*

Table S1. Length and width of each strain.

**Strain**	**Length[a] (μm)**	**N[b]**	***p* value[c]**
RU1651 (WT)	2.76 ± 0.79	346	NA
RU1724 (*sanA*^{M27R})	3.02 ± 1.02	389	0.0002 (vs WT)
RU1865 (Δ*sanA*)	3.00 ± 1.01	313	0.0008 (vs WT)
RU1635 (Δ*rodZ*)	4.22 ± 1.53	194	NA
RU1636 (Δ*rodZ sanA*^{M27R})	3.54 ± 1.09	316	<0.0001 (vs Δ*rodZ*)
RU1949 (Δ*rodZ* Δ*sanA*)	3.80 ± 1.20	307	0.0006 (vs Δ*rodZ*)
**Strain**	**Width[a] (μm)**	**N[b]**	***p* value[c]**
RU1651 (WT)	0.88 ± 0.06	346	NA
RU1724 (*sanA*^{M27R})	0.85 ± 0.06	389	<0.0001 (vs WT)
RU1865 (Δ*sanA*)	0.88 ± 0.07	313	0.58 (vs WT)
RU1635 (Δ*rodZ*)	2.03 ± 0.59	194	NA
RU1636 (Δ*rodZ sanA*^{M27R})	1.69 ± 0.39	316	<0.0001 (vs Δ*rodZ*)
RU1949 (Δ*rodZ* Δ*sanA*)	1.67 ± 0.35	307	<0.0001 (vs Δ*rodZ*)

[a]Mean length and width ± standard deviation (SD) are shown.

[b]Nubmers of cells counted are shown.

[c]*p* values were determined by unpaired *T* test. Statistical significance was defined as $P < 0.05$.

Table S2. Strains used in this study.

**Strain**	**Relevant genotypes**	**Reference**
BW25113	WT	(Baba *et al.*, 2006)
RU2	BW25113 Δ*rodZ::kan*	(Shiomi *et al.*, 2008)
RU383	BW25113 *sfgfp-rodZ*	(Ikebe *et al.*, 2018)
RU1353	BW25113 *sfgfp-rmr*	(Ago *et al.*, 2023)
RU1478	RU1353 *sanA*^{M27R} (original suppressor of RU1353)	This study
RU1601	BW25113 *sfgfp-rodZ* Δ*rlpA*::cat *mrdAT52I*	(Ago *et al.*, 2023)
RU1651	BW25113 Δ*cdd*::cat	This study
RU1652	RU1478 Δ*cdd*::cat	This study
RU1665	BW25113 *sfgfp-rodZ* Δ*yhdE::cat mreB*^{A125V}	(Ago *et al.*, 2023)
RU1695	BW25113 *sfgfp-rodZ* Δ*cdd::cat sanA*^{M27R}	This study
RU1696	BW25113 *sfgfp-rmr* Δ*cdd::cat sanA*^{M27R}	This study
RU1724	BW25113 Δ*cdd::cat sanA*^{M27R}	This study
C41(DE3)	a derivative strain of BL21(DE3) for protein overproduction	(Miroux and Walker, 1996)
RU1865	BW25113 Δ*cdd::cat* Δ*sanA::kan*	This study
RU1861	BW25113 Δ*sanA*::*kan*	This study
RU1635	BW25113 Δ*cdd::cat* Δ*rodZ::kan*	This study
RU1636	BW25113Δ*cdd::cat sanA*^{M27R} Δ*rodZ::kan*	This study
RU1933	BW25113Δ*cdd* Δ*sanA*	
RU2994	BW25113Δ*cdd* Δ*sanA* Δ*rodZ::kan*	This study
RU1934	BW25113 Δ*sanA* (RU1861 kan^{S})	This study
RU1949	BW25113 *sfgfp-rmr* Δ*cdd::cat* Δ*sanA::kan*	This study
RU1965	BW25113 Δ*mrcA::kan*	This study
RU1998	BW25113 Δ*mrcA*	This study
RU1966	BW25113 Δ*mrcB::kan*	This study
RU1999	BW25113 Δ*mrcB*	This study
RU2091	BW25113 Δ*mrcA* Δ*sanA::kan*	This study
RU2110	BW25113 Δ*mrcA* Δ*sanA*	This study
RU2092	BW25113 Δ*mrcB* Δ*sanA::kan*	This study

RU2112	BW25113 Δ*mrcB* Δ*sanA*	This study
RU2203	BW25113 Δ*lpoA::kan*	This study
RU2208	BW25113 Δ*lpoA*	This study
RU2204	BW25113 Δ*lpoB::kan*	This study
RU2209	BW25113 Δ*lpoB*	This study
RU2205	BW25113 Δ*sanA* Δ*lpoA::kan*	This study
RU2210	BW25113 Δ*sanA* Δ*lpoA*	This study
RU2206	BW25113 Δ*sanA* Δ*lpoB::kan*	This study
RU2211	BW25113 Δ*sanA* Δ*lpoB*	This study
RU2979	BW25113 *mrcB-3 x flag*::*cat*	This study
RU2993	BW25113 *mrcB-3 x flag*	This study
MG1655	WT	Lab stock
RU2879	MG1655 Δ*sanA::kan*	This study
RU2880	MG1655 Δ*elyC::kan*	This study
JW2132*	Δ*sanA::kan*	(Baba *et al.*, 2006)
JW3359*	Δ*mrcA::kan*	(Baba *et al.*, 2006)
JW0145*	Δ*mrcB::kan*	(Baba *et al.*, 2006)
JW3116*	Δ*lpoA::kan*	(Baba *et al.*, 2006)
JW5157*	Δ*lpoB::kan*	(Baba *et al.*, 2006)
JW0903	Δ*elyC::kan*	(Baba *et al.*, 2006)
SN208	MG1655 *panD-3xflag::cat*	(Nozaki *et al.*, 2012)
DHM1	a strain for bacterial two-hybrid assay	(Karimova *et al.*, 2005)

* These strains were obtained from National BioResource Project (https://shigen.nig.ac.jp/ecoli/strain/).

Table S3. Primers used in this study.

Primer	Sequence
sanA-up100 (1865)	TCCTGGTGTCGAAATTCCCG
sanA-down100 (1866)	TCAATCGCGCCTGCGCGCCA
sanA-f (BamHI) (1874)	GCGGATCCGTTAAAGCGCGTGTTCCTCAG
sanA-r (EcoRI) (1875)	GCGAATTCCTACTTTCCTTGTTTCTTTTG
ccd-H1 (1895)	ACGGGTT CGTAAACTGT TATCCCATTA CATGATTATG AGGCAACGCCATGGTGTAGGCTGGAGCTGCTTC
ccd-H2 (1896)	GATTGCAAGTTATCCGCAAGTTGGGCAAAAGCGGTT TGAAAACGTGGATGCATATGAATATCCTCCTTA
mrcA-f (BamHI) (2145)	GCGGATCCCAAGTTCGTAAAGTATTTTTTG
mrcA-r (EcoRI) (2146)	GCGAATTCTCAGAACAATTCCTGTGCCT
mrcB-f (BamHI) (2147)	GCGGATCCCGCCGGGAATGACCGCGAGCC
mrcB-r (EcoRI) (2148)	GCGAATTCTTAATTACTACCAAACATATCC
pKT25-*sanA*_R (2450)	TATATCGATGAATTCCTACTTTCCTTGTTTCTTTTG
pUT18C-*sanA-D174*_F (2402)	GACTCTAGAGGATCCGTTAAAGCGCGTGTTCCTCAG
pUT18C-*sanA*_R (2451)	TATATCGATGAATTCCTACTTTCCTTGTTTCTTTTG
mrcB-R190A-f (2437)	GGAGAACAACGCTCAGTTCG
mrcB-R190A-r (2438)	CGAACTGAGCGTTGTTCTCC
mrcB-R196A-f (2441)	CGGTTTCTTCGCTCTTGATC
mrcB-R196A-r (2442)	GATCAAGAGCGAAGAAACCG
mrcB-E211A-f (2364)	GCCAAACGGTGCGCAGCGTC
mrcB-E211A-r (2365)	GACGCTGCGCACCGTTTGGC
mrcB-D321A-f (2366)	AGCGGCGCCAACGAAATCCG
mrcB-D321A-r (2367)	CGGATTTCGTTGGCGCCGCT
pKT25(pUT18C)-*mrcB*_F (2447)	GACTCTAGAGGATCCCGCCGGGAATGACCGCGAGCC
pKT25-*mrcB*_R (2448)	ACGACGGCCGAATTCTTAATTACTACCAAACATATC
pUT18C-*mrcB*_R (2449)	TATATCGATGAATTCTTAATTACTACCAAACATATC
pKT25_Fcomp (2400)	GGATCCTCTAGAGTCGACCC
pKT25_Rcomp (2401)	GAATTCGGCCGTCGTTTTAC

pUT18C_Fcomp (2404)	GGATCCTCTAGAGTCGACCTG
pUT18C_Rcomp (2405)	GAATTCATCGATATAACTAAG
sanA-f (NcoI) (1926)	GGCCATGGTAAAGCGCGTGTTCC
sanA-r non-stop (XhoI) (1927)	GCCTCGAGCTTTCCTTGTTTCTTTTGTAATTC
pET28a-Amp-FLAG-f (2514)	CTTTCGGGCTTTGGGTACCTTACTCGAGAAGCTTGACTTTGTC
pET28a-Amp-FLAG-r (2515)	GACAAAGTCAAGCTTCTCGAGTAAGGTACCCAAAGCCCGAAAG
pET28a-Amp-FLAG-*mrcB(M46-)*_F (2394)	AGGAGATATACCATGGACTACAAGGACGACGATGACAAAGTCAAGCTTCTCGAGATGCCGCGCAAAGGTAAGGG
pET28a-Amp-FLAG-*mrcB(M46-)*_R (2395)	CGGGCTTTGGGTACCTTAATTACTACCAAACATATCC
pET28a_Fcomp (2396)	CATGGTATATCTCCTTCTTAAAG
pET28a_Rcomp (2397)	GGTACCCAAAGCCCGAAAGG
sanA-f (EcoRI) (1872)	GCGAATTCTTAAAGCGCGTGTTCCTCAG
sanA-r (HindIII) (1873)	CGAAGCTTCTACTTTCCTTGTTTCTTTTG
sanA-f(NcoI) (2064)	GCCCATGGTAAAGCGCGTGTTCCTCAGC
sanA-r(XbaI) (2093)	GCTCTAGACTACTTTCCTTGTTTCTTTTG
pBAD33-MCS3-*mrcA*_R (2389)	AAAACAGCCTCTAGATCAGAACAATTCCTGTGCCTC
pBAD33-MCS3-*mrcB*_F (2390)	ATTCACCATGGTACCATGGCCGGGAATGACCGCGAG
pBAD33-MCS3-*mrcB*_R (2391)	AAAACAGCCTCTAGATTAATTACTACCAAACATATCC
pBAD33-MCS3_Fcomp (2392)	GGTACCATGGTGAATTCCTC
pBAD33-MCS3_Rcomp (2393)	TCTAGAGGCTGTTTTGGCGG
PBP1B-FLAG-H1 (3223)	AAGACAGCGACGGTGTAGCCGGTTGGATCAAGGATATGTTTGGTAGTAATGACTACAAGGATCATGATGG
PBP1B-FLAG-H2 (3224)	TGTTATTTTACCGGATGGCAACTCGCCATCCGGTATTTCACGCTTAGATGCATATGAATATCCTCCTTAG
pET-Duet-sanA-His.FOR (3225)	AACTTTAAGAAGGAGATATACCATGGTAAAGCG
pET-Duet-SanA-His.REV (3226)	TACGATTACTTTCTGTTCGATCAGTGGTGGTGGTGGTGGTGC
pET-Duet-MCS1.FOR (3227)	TCGAACAGAAAGTAATCGTATTGTACACG

pET-Duet-MCS1.REV (3228)	TATATCTCCTTCTTAAAGTTAAACAAAATTATTTCTAGAGGGGA
pET-Duet-PBP1Bperi.FOR (3229)	TATAAGAAGGAGATATACATATGGACTACAAGGACGACGATGAC
pET-Duet-PBP1Bperi.REV (3230)	TTGCTCAGCGGTGGCAGCAGTTAATTACTACCAAACATATCCTTGATCC
pET-Duet-MCS2.FOR (3231)	CTGCTGCCACCGCTGAG
pET-Duet-MCS2.REV (3232)	ATGTATATCTCCTTCTTATACTTAACTAATATACTAAGATGGGGAA
pBAD-FRT cloning.FOR (3243)	GAGGAATTCACCATGGTACCATGGTGgAGGCTGGAGC
pBAD-FRT cloning.REV (3244)	GCCTGCAGGTCGACTCTAGATTAATTACTACCAAAC
pBAD33.FOR (3245)	TCTAGAGTCGACCTGCAGGCATGCAAGC

Table S4. Plasmids used in this study.

Plasmid	Genes, vector, antibiotics resistance	Reference
pKT25	P_{lac}::T25, Kan^R	(Karimova *et al.*, 1998)
pUT18C	P_{lac}::T18, Amp^R	(Karimova *et al.*, 1998)
pRU1697	*sanA* in pKT25, Kan^R	This study
pRU1623	*sanA* in pUT18C, Amp^R	This study
pRU2105	*mrcA* in pKT25, Kan^R	This study
pRU2106	*mrcB* in pKT25, Kan^R	This study
pDS1271	*rodZ* in pKT25, Kan^R	(Yoshii *et al.*, 2019)
pDS1266	*rodZ* in pUT18C, Amp^R	(Yoshii *et al.*, 2019)
pDS1274	*rmr* in pKT25, Kan^R	(Ago *et al.*, 2023)
pDS1269	*rmr* in pUT18C, Amp^R	(Yoshii *et al.*, 2019)
pET28Ap	pET28a derivative, Amp^R	(Nozaki *et al.*, 2012)
pRU1693	*sanA-his$_6$* in pET28Ap, Amp^R	This study
pRU2417	*flag-mrcB$^{M46\text{-}N844}$* in pET28Ap, Amp^R	This study
pET-duet	pBR322 derivative, Amp^R	Lab stock
pRU2980	*sanA-his$_6$* in pET-duet, Amp^R	This study
pRU2981	*flag-mrcB$^{M46\text{-}N844}$* in pET-duet, Amp^R	This study
pRU2982	*sanA-his$_6$/ flag-mrcB$^{M46\text{-}N844}$* in pET-duet, Amp^R	This study
pDSW207	P_{trc}–*gfp* pBR322 derivative, Amp^R	(Weiss *et al.*, 1999)
pDSW208	pBR322 derivative P_{trc}–*gfp*, Amp^R	(Weiss *et al.*, 1999)
pRU1565	pDSW208-Δ*gfp*, Amp^R	This study
pRU1582	*sanA* in pRU1565, Amp^R	This study
pRU1825	pTrc99A derivative, Kan^R	(Hayashi *et al.*, 2024)
pRU1964	*sanA* in pRU1825	This study
pBAD24	pBR322 derivative containing P_{BAD} promoter, Amp^R	(Guzman *et al.*, 1995)
pBAD33	pACYC184 derivative containing P_{BAD} promoter, Cm^R	(Guzman *et al.*, 1995)
pRU1276	pBAD33-MCS3, Cm^R	This study
pRU2416	*mrcB* in pRU1276, Cm^R	This study
pRU2998	*mrcB-3 x flag* in pRU1276, Cm^R	This study
pKD46	Lambda Red recombinase, Amp^R	(Datsenko and Wanner, 2000)
pKD3	FRT-cat-FRT, Cm^R Amp^R	(Datsenko and Wanner, 2000)

pCP20	yeast Flp recombinase gene, Cm^R	(Datsenko and Wanner, 2000)

Legend for supplementary Figures

Fig. S1 CPRG assay of WT, Δ*sanA*, and Δ*elyC* cells. Cells of the indicated deletion mutants in an MG1655 strain background were streaked on CPRG indicator agar (20 mg/ml CPRG and 50 mM IPTG). The plates were incubated for 24 h at 30, 37, or 43°C.

Fig. S2 Sensitivity of WT and Δ*sanA* cells to antibiotics. An overnight culture of the indicated strains was diluted serially (from 10^{-1} to 10^{-6}) and spotted onto L plates containing the indicated antibiotics. The plates were incubated at 37°C or 43˚C for 24 h.

Fig. S3 Sensitivity of SanA-overproducing cells to antibiotics. An overnight culture of WT cells carrying a vector (pRU1825) or a plasmid encoding *sanA* (pRU1964) was diluted serially (from 10^{-1} to 10^{-6}) and spotted onto L plates containing the indicated antibiotics in the presence of IPTG. The plates were incubated at 37°C or 43˚C for 24 h.

Fig. S4 Comparison between BW25113 (WT) and RU1934 (Δ*sanA*). Growth curve (A) and phase contrast images (B) of BW25113 and RU1934. Scale bars: 2 µm.

Fig. S5 Rod complex in RU383 (WT; sfGFP-RodZ), RU1353 (RMR; sfGFP-RMR), RU1696 (sfGFP-RMR SanA^{M27R}). Violin plots showing the distribution of the size of the Rod complex estimated from images of sfGFP-RodZ or sfGFP-RMR in the indicated strains. Average and standard deviation (S.D.) are shown. *p* Values were determined by unpaired *T* test.

Fig. S6 The effects of SanA^{M27R} mutant and deletion of the *sanA* gene in ΔrodZ cells. (A) Growth curve of RU1651 (WT), RU1724 (*sanA^{M27R}*), RU1865 (Δ*sanA*), RU1635 (Δ*rodZ*), RU1636 (Δ*rodZ sanA^{M27R}*), and RU2994 (Δ*rodZ* Δ*sanA*) cells. The cells were cultured in L medium at 37 °C and their absorbance (OD_{660}) was recorded every 5 min. (B) Morphology of RU1651 (WT), RU1724 (*sanA^{M27R}*), RU1865 (Δ*sanA*), RU1635 (Δ*rodZ*), RU1636 (Δ*rodZ sanA^{M27R}*), and RU2994 (Δ*rodZ* Δ*sanA*) cells cultured in L medium to log-phase at 37 °C. Scale bar: 2 µm.

Fig. S7 Sensitivity of Δ*sanA* or *sanA^{M27R}* cells to mecillinam and aztreonam. An overnight culture of the indicated strains was diluted serially (from 10^{-1} to 10^{-6}) and spotted onto L plates containing the indicated antibiotics. The plates were incubated at 37°C or 43˚C for 24 h.

Fig. S8 Interaction between SanA and PBP1B. The interaction between SanA and PBP1B was examined using coimmunoprecipitation. Cell lysates containing SanA-His6 and FLAG-PBP1B$^{M46\text{-}N844}$ were mixed with the Ni-NTA agarose. The FLAG-PBP1BM46-N844 was eluted by imidazole. The samples were separated using SDS-PAGE (12%), and western blotting was performed with anti-FLAG or anti- SanA antibodies. The blot with anti-FLAG M2 antibody is shown on the bottm while the blot with anti- SanA antibody are shown in the top. I: input; UB: unbound (supernatant of the centrifugation after the mixing of the lysates and Ni-NTA agarose); W1–W3: washed samples (supernatant of the centrifugation after washing of N-NTA agarose containing the indicated concentration of imidazole).

Fig. S9 Protein levels of SanA. Immunoblot of RU1934 (Δ*sanA*) and BW25113 carrying pRU1565 (vec) and pRU1582 (*sanA*) cells grown in the presence (+) and absence (-) of 1 mM IPTG with the anti-SanA. The sample of BW25113 carrying pRU1582 grown in the presence of IPTG was diluted 100-fold. The numbers shown below the gel indicate relative SanA levels, with the amount in BW25113 harboring pRU1565 (vec) cultured without IPTG defined as 1. Thus, SanA expressed from pRU1582 in the presence of 1 mM IPTG was approximately 1,500-fold higher than that expressed from the chromosomal copy.

Fig. S10 Morphology of cells overproducing SanA. WT cells carrying a vector or a plasmid encoding *sanA* were grown in L or NBMSM in the presence or absence of IPTG. Scale bar: 2 μm.

Fig. S11 Properties of PBP1B-3xFLAG. (A) Δ*mrcA::kan* was transduced to WT (BW25113), RU1999 (Δ*mrcB*) or RU2993 (*mrcB-3xflag*) cells by P1 transduction. Plates were incubated at 37˚C for 24 h. (B) Immunoblots of BW25113 (WT), RU2993 (*mrcB-3x flag*: 1B-3F) and BW25113 carrying pRU1276 (vec) and pRU2998(*mrcB-3x flag*: 1B-3F) cells grown for 2 h (top) or 24 h (bottom) at 37˚C in the presence (+) and absence (-) of 0.001% arabinose with the anti-FLAG antibody. The numbers shown below the gels indicate relative PBP1B-3xFLAG levels, with the amount in RU2993 defined as 1.

Fig. S12 Cell shape of cells overproducing SanA and LpoB. WT cells carrying plasmids encoding *sanA* whose expression is induced by IPTG and *lpoB* whose expression is induced by arabinose were grown in L-medium in the presence or absence of IPTG or arabinose at 37˚C for 24h. (B) Roundness of each cell. The average and

standard deviation are shown. *p* values were determined by an unpaired *T*-test. n.s.: $p>0.05$

Fig. S13 Growth curve and morpholgy of cells producing PBP1B. Growth curve (A) and phase contrast images (B) of RU383 and RU1353 carrying pRU1276 (vec) or pRU2416 (*mrcB*). Scale bars: 2 µm.

Fig. S14 Relation between peptidoglycan synthesis and repair. (A) Δ*mrcA::kan* or Δ*mrcB::kan* was transduced to WT (RU383) or RU1353 (RMR) cells by P1 transduction. (B) Cell shape of cells overproducing SanA. WT (RU383), RU1665 ($MreB^{A125V}$), or RU1601 ($PBP2^{T52I}$) cells carrying a vector or a plasmid carrying *sanA* were grown in L-medium in the presence or absence of IPTG or arabinose at 37˚C for 24h.

Supplementary References

Ago, R., Tahara, Y.O., Yamaguchi, H., Saito, M., Ito, W., Yamasaki, K., *et al.* (2023) Relationship between the Rod complex and peptidoglycan structure in Escherichia coli. *MicrobiologyOpen* **12**: e1385.

Baba, T., Ara, T., Hasegawa, M., Takai, Y., Okumura, Y., Baba, M., *et al.* (2006) Construction of Escherichia coli K-12 in-frame, single-gene knockout mutants: the Keio collection. *Molecular systems biology* **2**: 2006.0008.

Datsenko, K.A., and Wanner, B.L. (2000) One-step inactivation of chromosomal genes in Escherichia coli K-12 using PCR products. *Proceedings of the National Academy of Sciences of the United States of America* **97**: 6640–6645.

Guzman, L.M., Belin, D., Carson, M.J., and Beckwith, J. (1995) Tight regulation, modulation, and high-level expression by vectors containing the arabinose PBAD promoter. *Journal of bacteriology* **177**: 4121–4130.

Hayashi, M., Takaoka, C., Higashi, K., Kurokawa, K., Margolin, W., Oshima, T., and Shiomi, D. (2024) Septal wall synthesis is sufficient to change ameba-like cells into uniform oval-shaped cells in Escherichia coli L-forms. *Commun Biol* **7**: 1569.

Ikebe, R., Kuwabara, Y., Chikada, T., Niki, H., and Shiomi, D. (2018) The periplasmic disordered domain of RodZ promotes its self-interaction in Escherichia coli. *Genes Cells* **23**: 307–317.

Karimova, G., Dautin, N., and Ladant, D. (2005) Interaction network among Escherichia coli membrane proteins involved in cell division as revealed by bacterial two-hybrid analysis. *Journal of bacteriology* **187**: 2233–2243.

Karimova, G., Pidoux, J., Ullmann, A., and Ladant, D. (1998) A bacterial two-hybrid system based on a reconstituted signal transduction pathway. *Proceedings of the National Academy of Sciences of the United States of America* **95**: 5752–5756.

Miroux, B., and Walker, J.E. (1996) Over-production of proteins in Escherichia coli: mutant hosts that allow synthesis of some membrane proteins and globular proteins at high levels. *J Mol Biol* **260**: 289–98.

Nozaki, S., Webb, M.E., and Niki, H. (2012) An activator for pyruvoyl-dependent l-aspartate α-decarboxylase is conserved in a small group of the γ-proteobacteria including Escherichia coli. *MicrobiologyOpen* **1**: 298–310.

Shiomi, D., Sakai, M., and Niki, H. (2008) Determination of bacterial rod shape by a novel cytoskeletal membrane protein. *EMBO J* **27**: 3081–3091.

Weiss, D.S., Chen, J.C., Ghigo, J.M., Boyd, D., and Beckwith, J. (1999) Localization of FtsI (PBP3) to the septal ring requires its membrane anchor, the Z ring, FtsA, FtsQ, and FtsL. *Journal of bacteriology* **181**: 508–520.

Yoshii, Y., Niki, H., and Shiomi, D. (2019) Division-site localization of RodZ is required for efficient Z ring formation in Escherichia coli. *Mol Microbiol* **111**: 1229–1244.

Fig. S1 Yamaguchi et al.,

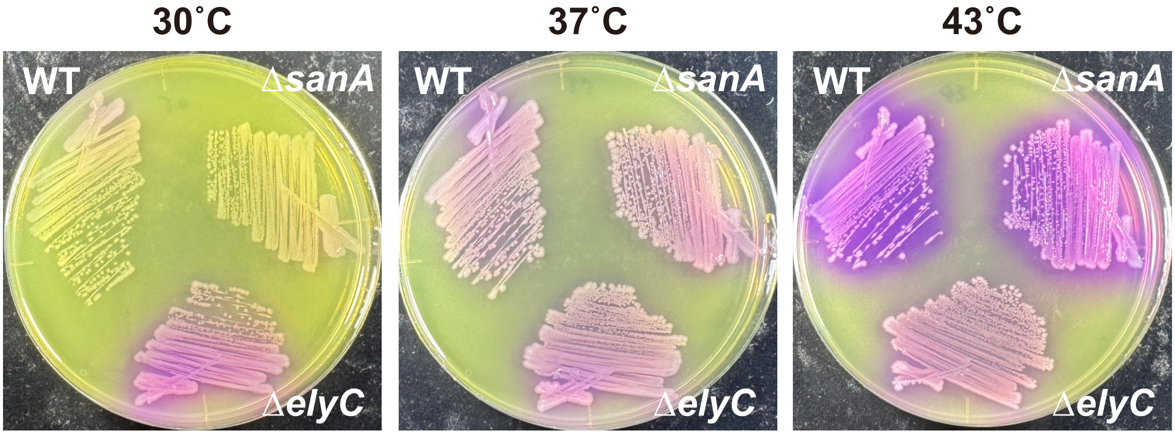

Fig. S1 CPRG assay of WT, ∆*sanA*, and ∆*elyC* cells. Cells of the indicated deletion mutants in an MG1655 strain background were streaked on CPRG indicator agar (20 mg/ml CPRG and 50 mM IPTG). The plates were incubated for 24 h at 30, 37, or 43°C.

Fig. S2 Yamaguchi et al.,

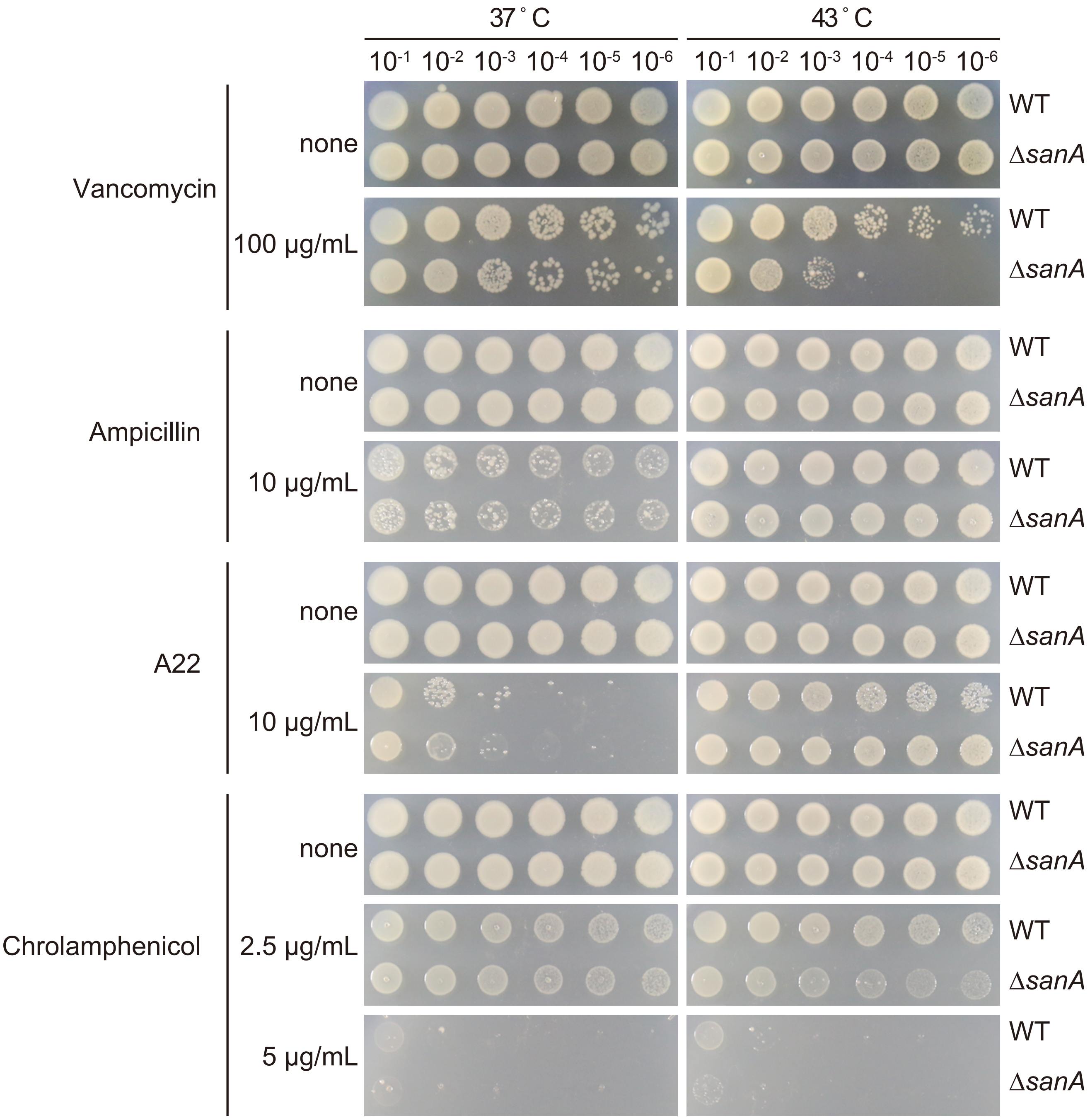

Fig. S2 Sensitivity of WT and Δ*sanA* cells to antibiotics. An overnight culture of the indicated strains was diluted serially (from 10^{-1} to 10^{-6}) and spotted onto L plates containing the indicated antibiotics. The plates were incubated at 37°C or 43˚C for 24 h.

Fig. S3 Yamaguchi et al.,

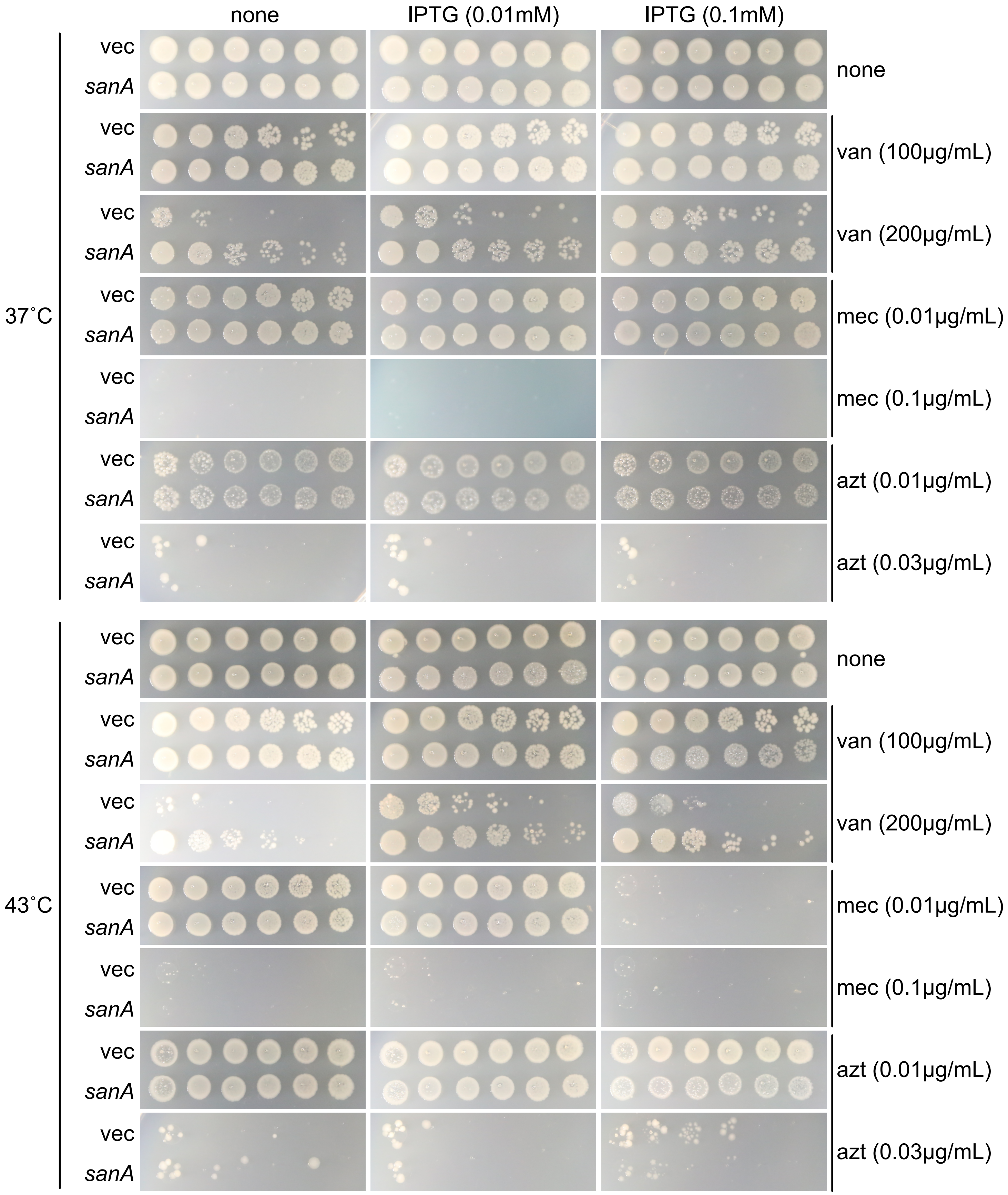

Fig. S3 Sensitivity of SanA-overproducing cells to antibiotics. An overnight culture of WT cells carrying a vector (pRU1825) or a plasmid encoding *sanA* (pRU1964) was diluted serially (from 10^{-1} to 10^{-6}) and spotted onto L plates containing the indicated antibiotics in the presence of IPTG. The plates were incubated at 37°C or 43˚C for 24 h.

Fig. S4 Yamaguchi et al.,

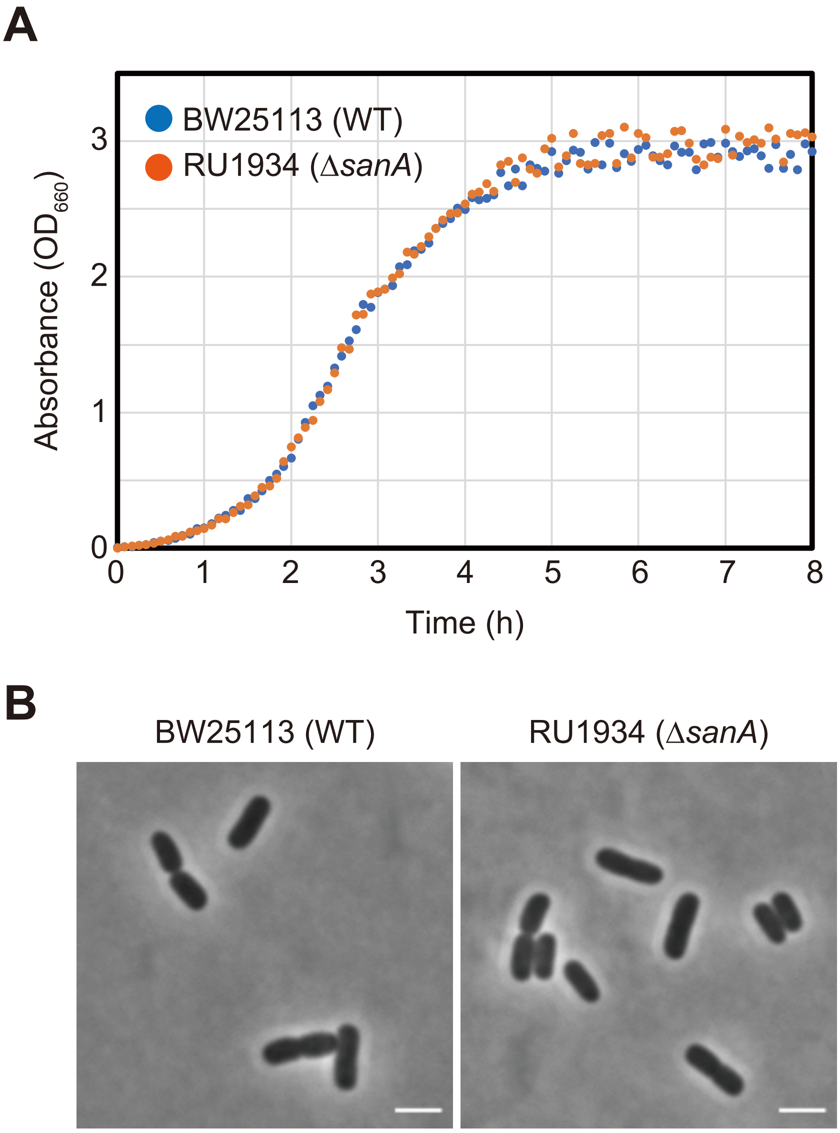

Fig. S4 Comparison between BW25113 (WT) and RU1934 (Δ*sanA*). Growth curve (A) and phase contrast images (B) of BW25113 and RU1934. Scale bars: 2 μm.

Fig. S5 Yamaguchi et al.,

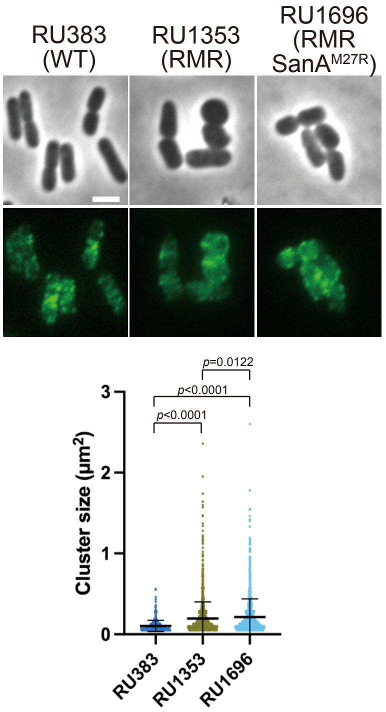

Fig S5 Rod complex in RU383 (WT; sfGFP-RodZ), RU1353 (RMR; sfGFP-RMR), RU1696 (sfGFP-RMR SanA^{M27R}). Violin plots showing the distribution of the size of the Rod complex estimated from images of sfGFP-RodZ or sfGFP-RMR in the indicated strains. Average and standard deviation (S.D.) are shown. *p* Values were determined by unpaired *T* test.

Fig. S6 Yamaguchi et al.,

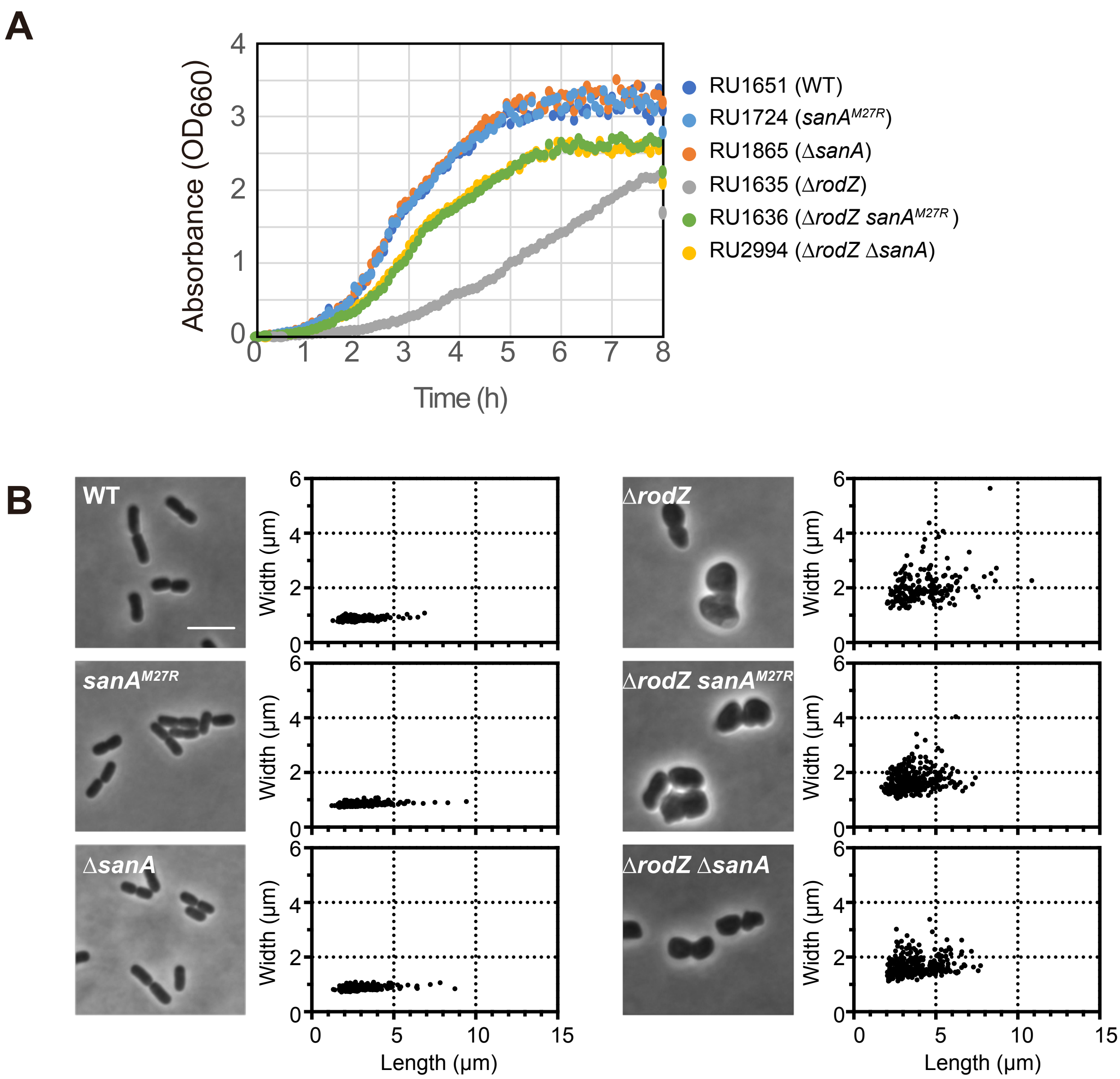

Fig. S6 The effects of SanA^{M27R} mutant and deletion of the *sanA* gene in Δ*rodZ* cells. (A) Growth curve of RU1651 (WT), RU1724 (*sanA^{M27R}*), RU1865 (Δ*sanA*), RU1635 (Δ*rodZ*), RU1636 (Δ*rodZ sanA^{M27R}*), and RU2994 (Δ*rodZ* Δ*sanA*) cells. The cells were cultured in L medium at 37 °C and their absorbance (OD_{660}) was recorded every 5 min. (B) Morphology of RU1651 (WT), RU1724 (*sanA^{M27R}*), RU1865 (Δ*sanA*), RU1635 (Δ*rodZ*), RU1636 (Δ*rodZ sanA^{M27R}*), and RU2994 (Δ*rodZ* Δ*sanA*) cells cultured in L medium to log-phase at 37 °C. Scale bar: 2 μm.

Fig. S7 Yamaguchi et al.,

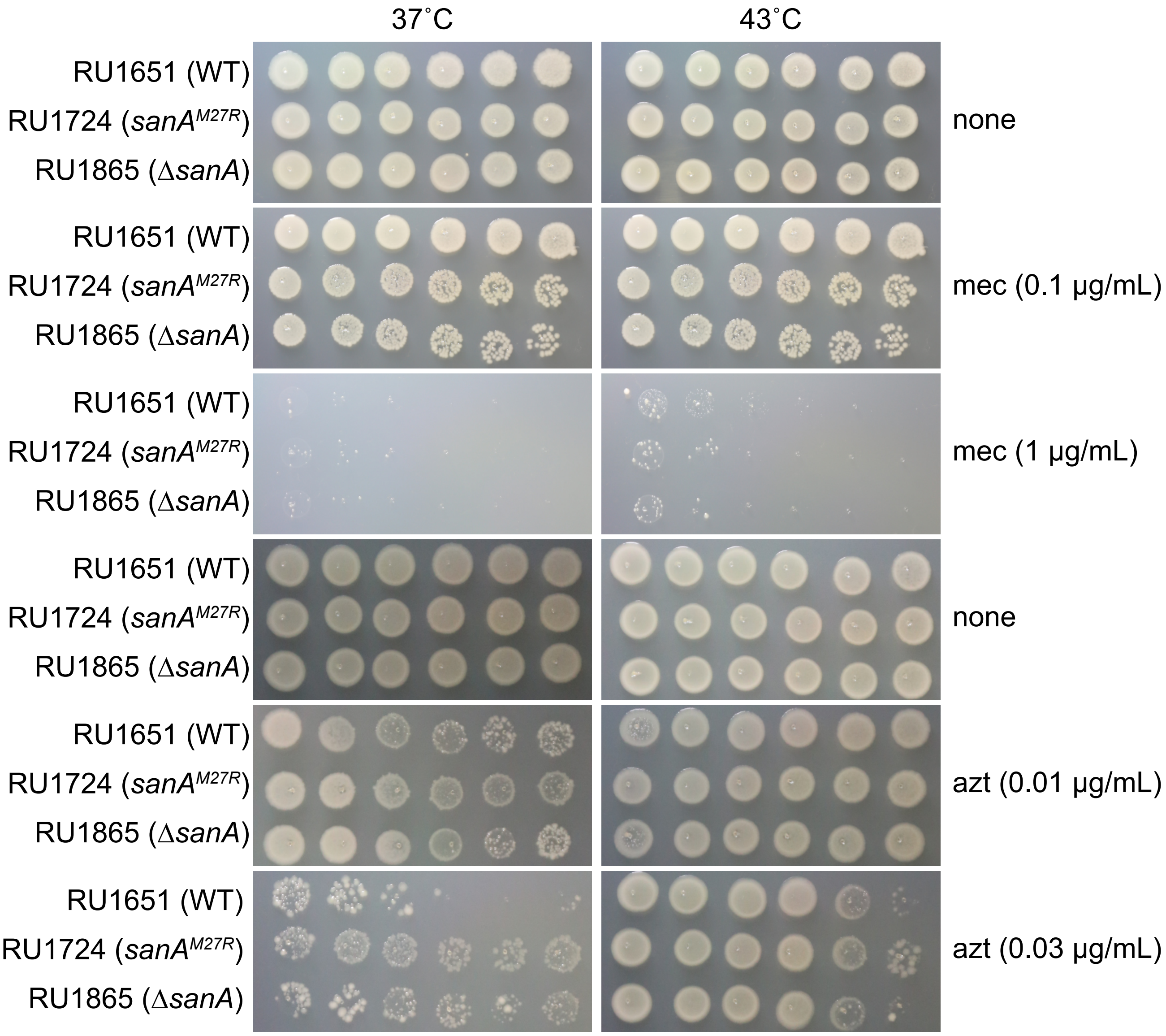

Fig. S7 Sensitivity of Δ*sanA* or *sanA*^{M27R} cells to mecillinam and aztreonam. An overnight culture of the indicated strains was diluted serially (from 10^{-1} to 10^{-6}) and spotted onto L plates containing the indicated antibiotics. The plates were incubated at 37˚C or 43˚C for 24 h.

Fig. S8 Yamaguchi et al.,

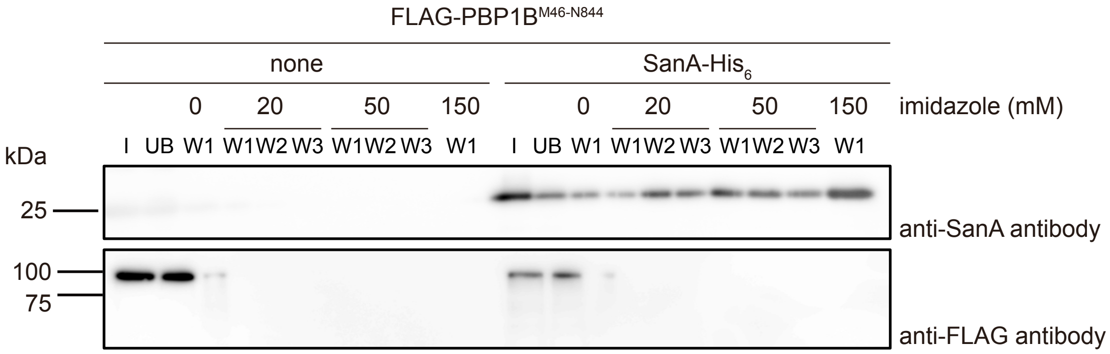

Fig. S8 Interaction between SanA and PBP1B. The interaction between SanA and PBP1B was examined using coimmunoprecipitation. Cell lysates containing SanA-His$_6$ and FLAG-PBP1B$^{M46-N844}$ were mixed with the Ni-NTA agarose. The FLAG-PBP1B$^{M46-N844}$ was eluted by imidazole. The samples were separated using SDS-PAGE (12%), and western blotting was performed with anti-FLAG or anti-SanA antibodies. The blot with anti-FLAG M2 antibody is shown on the bottm while the blot with anti-SanA antibody are shown in the top. I: input; UB: unbound (supernatant of the centrifugation after the mixing of the lysates and Ni-NTA agarose); W1–W3: washed samples (supernatant of the centrifugation after washing of N-NTA agarose containing the indicated concentration of imidazole).

Fig. S9 Yamaguchi et al.,

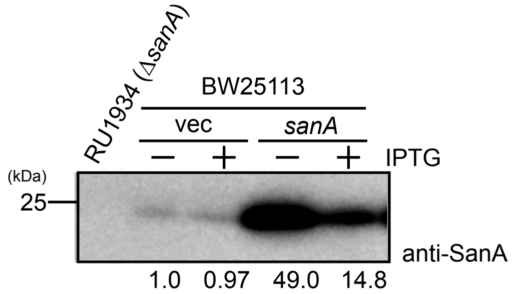

Fig. S9 Protein levels of SanA. Immunoblot of RU1934 (Δ*sanA*) and BW25113 carrying pRU1565 (vec) and pRU1582 (*sanA*) cells grown in the presence (+) and absence (-) of 1 mM IPTG with the anti-SanA. The sample of BW25113 carrying pRU1582 grown in the presence of IPTG was diluted 100-fold. The numbers shown below the gel indicate relative SanA levels, with the amount in BW25113 harboring pRU1565 (vec) cultured without IPTG defined as 1. Thus, SanA expressed from pRU1582 in the presence of 1 mM IPTG was approximately 1,500-fold higher than that expressed from the chromosomal copy.

Fig. S10 Yamaguchi et al.,

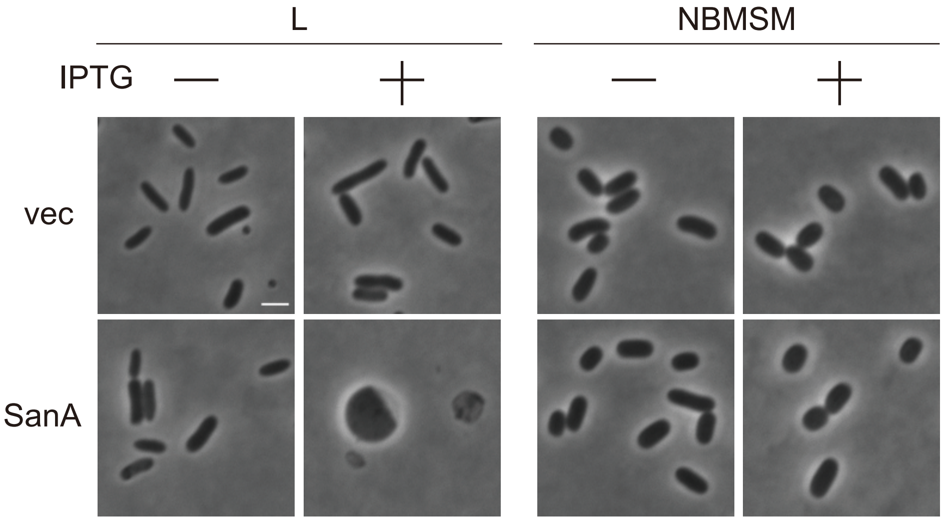

Fig. S10 Morphology of cells overproducing SanA. WT cells carrying a vector or a plasmid encoding *sanA* were grown in L or NBMSM in the presence or absence of IPTG. Scale bar: 2 μm.

Fig. S11 Yamaguchi et al.,

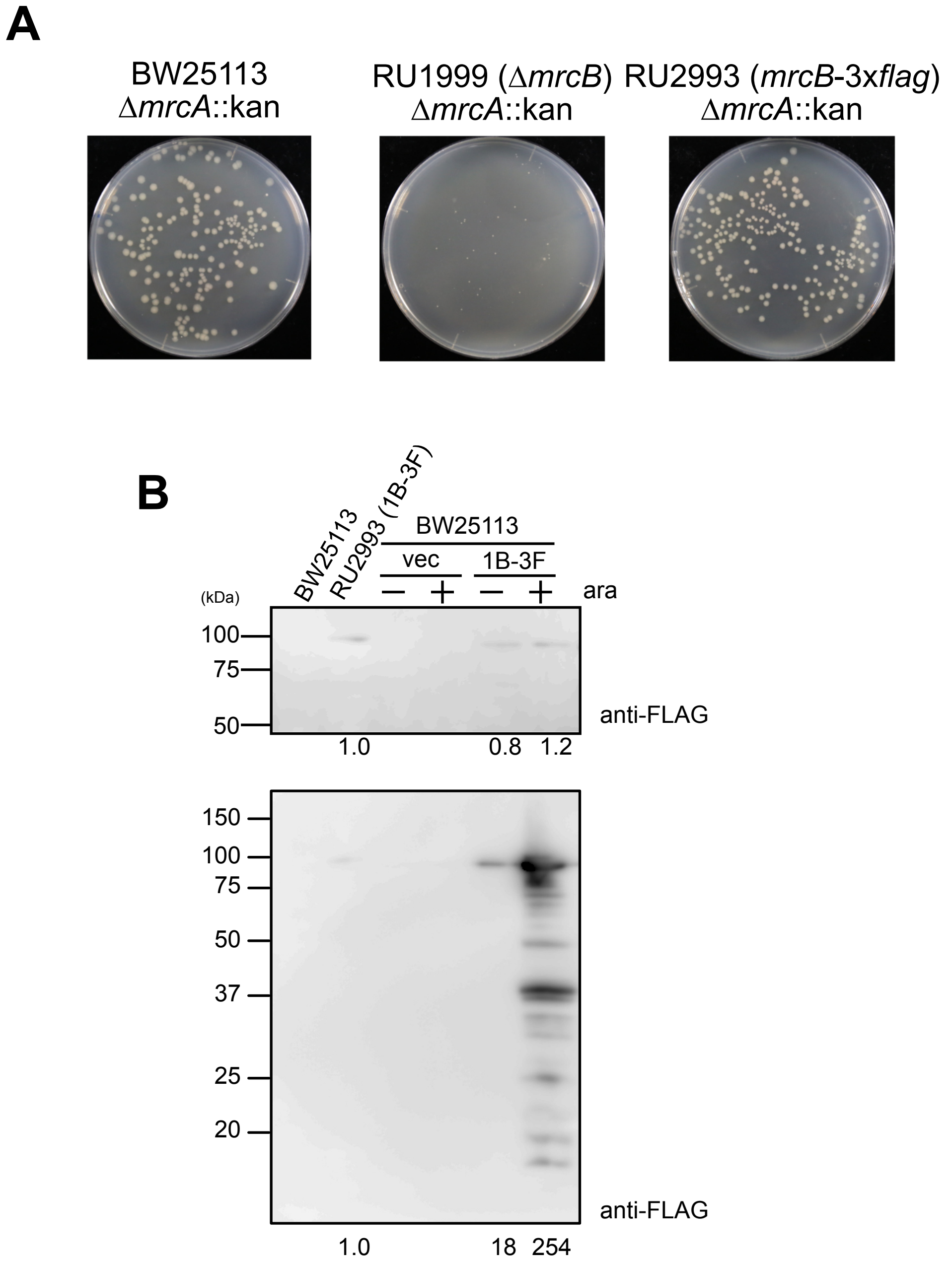

Fig. S11 Properties of PBP1B-3xFLAG. (A) Δ*mrcA::kan* was transduced to WT (BW25113), RU1999 (Δ*mrcB*) or RU2993 (*mrcB-3xflag*) cells by P1 transduction. Plates were incubated at 37˚C for 24 h. (B) Immunoblots of BW25113 (WT), RU2993 (*mrcB-3x flag*: 1B-3F) and BW25113 carrying pRU1276 (vec) and pRU2998(*mrcB-3x flag*: 1B-3F) cells grown for 2 h (top) or 24 h (bottom) at 37˚C in the presence (+) and absence (-) of 0.001% arabinose with the anti-FLAG antibody. The numbers shown below the gels indicate relative PBP1B-3xFLAG levels, with the amount in RU2993 defined as 1.

Fig. S12 Yamaguchi et al.,

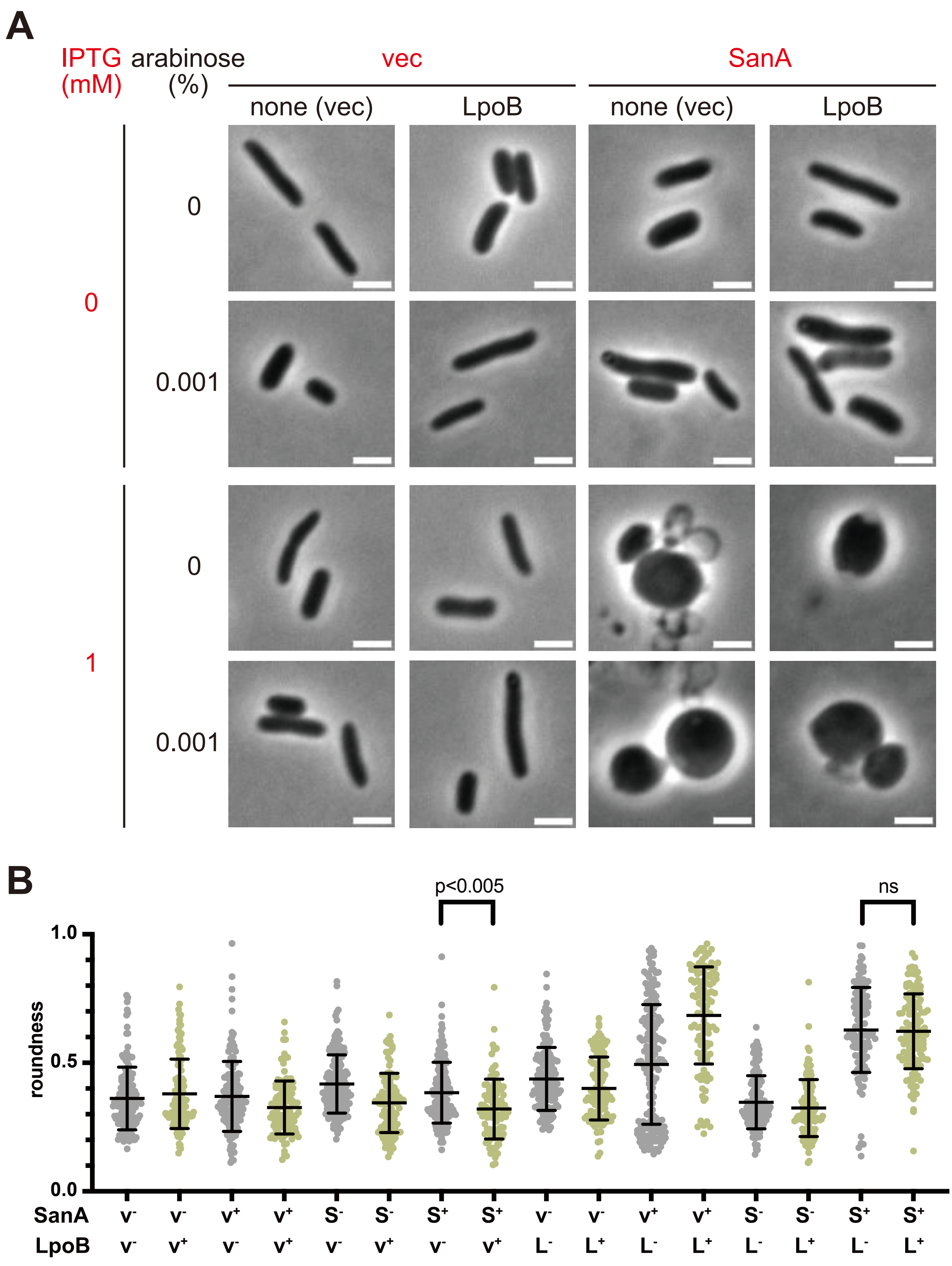

Fig. S12 Cell shape of cells overproducing SanA and LpoB. WT cells carrying plasmids encoding *sanA* whose expression is induced by IPTG and lpoB whose expression is induced by arabinose were grown in L-medium in the presence or absence of IPTG or arabinose at 37˚C for 24h. (B) Roundness of each cell. The average and standard deviation are shown. p values were determined by an unpaired T-test. n.s.: p>0.05. + and - indicate in the presence and absence of the inducer (1 mM IPTG or 0.001% arabinose), respectively.

Fig. S13 Yamaguchi et al.,

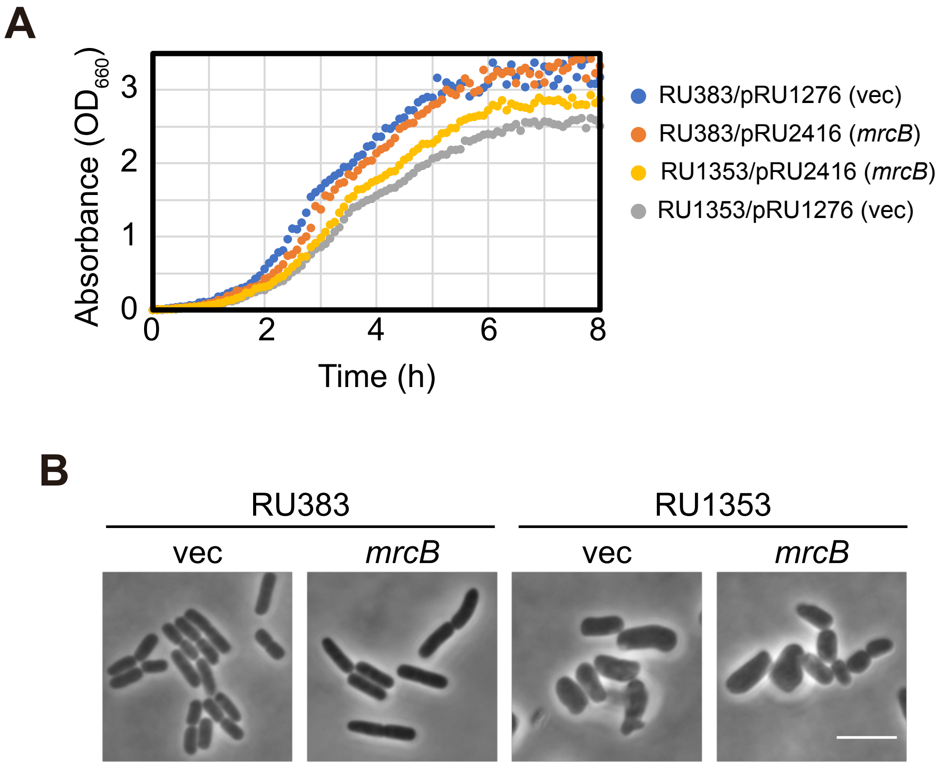

Fig. S13 Growth curve and morpholgy of cells producing PBP1B. Growth curve (A) and phase contrast images (B) of RU383 and RU1353 carrying pRU1276 (vec) or pRU2416 (*mrcB*). Scale bars: 2 μm.

Fig. S14 Yamaguchi et al.,

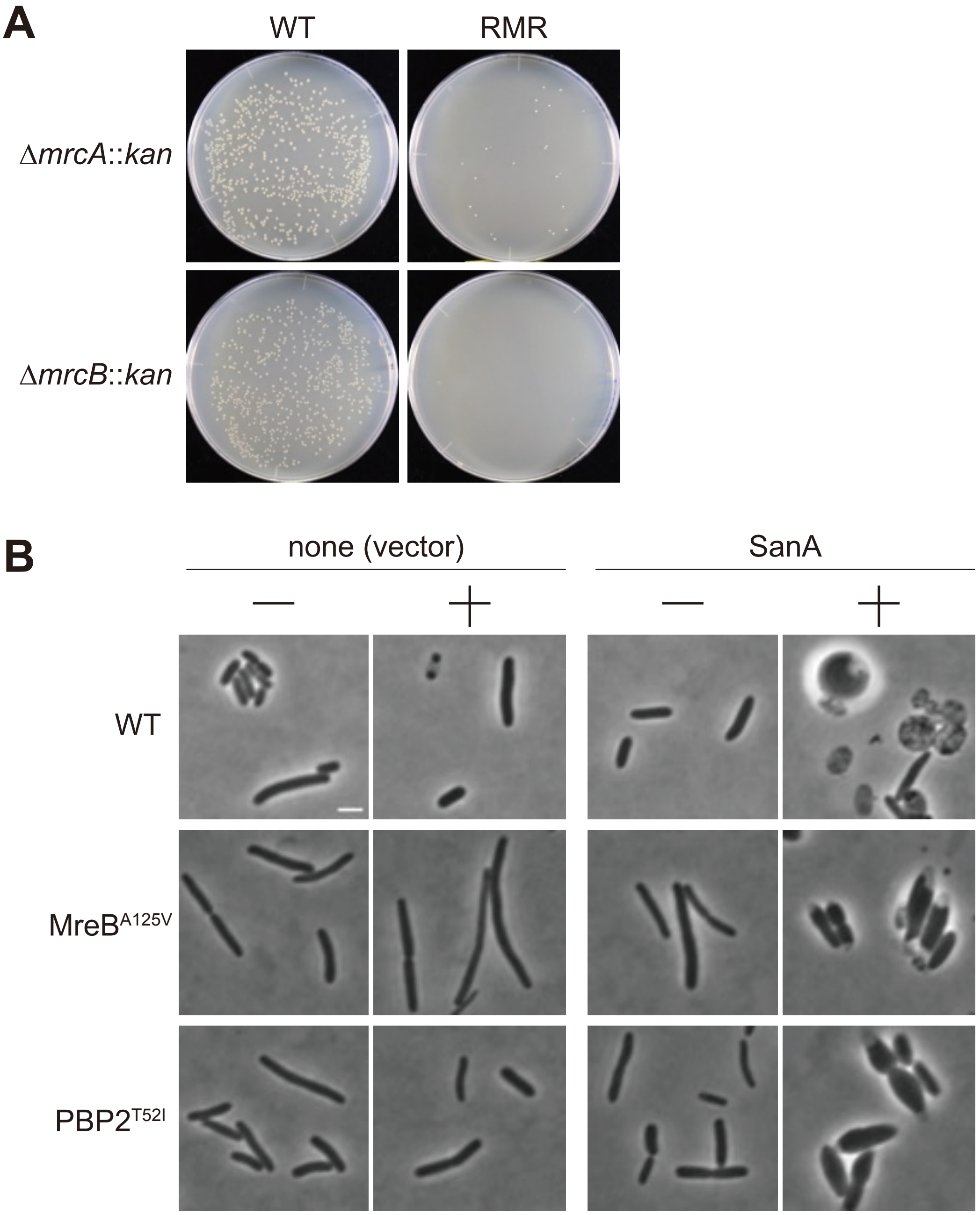

Fig. S14 Relation between peptidoglycan synthesis and repair. (A) Δ*mrcA::kan* or Δ *mrcB::kan* was transduced to WT (RU383) or RU1353 (RMR) cells by P1 transduction. (B) Cell shape of cells overproducing SanA. WT (RU383), RU1665 (MreB^{A125V}), or RU1601 (PBP2^{T52I}) cells carrying a vector or a plasmid carrying *sanA* were grown in L-medium in the presence or absence of IPTG or arabinose at 37˚C for 24h.
